# Barriers and facilitators to usability of a smartphone-based digital mental health tool in older adults: Insights from a secondary analysis of mindLAMP

**DOI:** 10.1016/j.inpsyc.2025.100123

**Published:** 2025-09-08

**Authors:** Elombe Calvert, Katherine Hackett, John Torous, Tania Giovannetti

**Affiliations:** a Department of Psychiatry, Beth Israel Deaconess Medical Center, Boston, MA, United States; b Department of Medicine, Icahn School of Medicine at Mount Sinai, New York, NY, United States; c Department of Psychology and Neuroscience, Temple University, Philadelphia, PA, United States

**Keywords:** Digital literacy, Digital mental health interventions, Digital divide, Access to care, Mental health apps

## Abstract

**Background:**

As demand for mental healthcare access grows among older adult populations, digital mental health tools have emerged as promising tools. However, bridging the digital divide among older technology users remains critical. This post-hoc analysis evaluated potential factors influencing the adoption of a digital mental health tool in older adults.

**Methods:**

We analyzed data from 37 older adults who used a digital phenotyping app (mindLAMP) for 4 weeks to capture passive sensor data and complete nightly surveys. We examined associations between baseline participant features including demographics, cognition, mood, technology attitudes and use, and usability outcomes including app training metrics, adherence, and self-reported usability.

**Results:**

Participants had a mean age of 72, with most identifying as female (68 %), college educated (76 %), retired (81 %), and White (59 %). The app demonstrated high usability, with baseline training averaging 20.2 (± 6.5) minutes and 80 % nightly survey completion. At study completion, 30/37 participants reported finding the app easy to use. While not significant after correction, female sex, Black race, and some college education emerged as potentially promising factors associated with better usability outcomes.

**Discussion:**

These findings suggest that with modest training, older adults can engage with digital health tools and report positive usability experiences. Differences in usability outcomes by sex, race, and education point to potential characteristics that may influence engagement. However, given the small, highly educated sample, these findings should be replicated in larger, more diverse cohorts to better understand which factors support the successful use of digital health tools in older adults.

## Introduction

Mental health disorders affect approximately 14 % of adults aged 60 and older, accounting for 10.6 % of total years lived with disability in this age group [1,2]. Despite this significant burden, older adults continue to face substantial barriers to accessing mental health care. Only half of individuals with mental health conditions receive treatment, and among adults over age 55, nearly 70 % do not engage with mental health services, often due to limited awareness of need, stigma, or discomfort with disclosure [3]. Digital mental health interventions, defined as psychosocial interventions delivered via digital platforms such as smartphones, wearable devices or the internet, have emerged as a promising strategy to expand access to care [4]. However, their suitability for older adult populations remains unclear due to digital barriers, including disparities in access, technological literacy, and trust in digital tools. To identify, understand, and ultimately reduce digital barriers, the current study examined relations between participant features and usability and adherence of a smartphone-based digital health tool over four weeks in a sample of older adults.

Among emerging digital mental health intervention approaches, smartphone-based digital phenotyping is a specific approach that offers novel opportunities for real-time monitoring of mental and cognitive health. Digital phenotyping is defined as the moment-by-moment quantification of human behavior using personal digital devices [5], to unobtrusively collect rich behavioral and physiological data in everyday life. Digital phenotyping apps that are available on personal smartphone devices can integrate passive data streams from sensors embedded in commercial smartphones (e.g., geolocation, accelerometry) with active cognitive and self-report assessments to provide both flexibility and depth to mental health research. While this approach has shown promise in adult psychiatric research contexts, its implementation in older adult cohorts has lagged behind younger populations.

Despite the growing availability of digital mental health interventions, a notable digital divide persists, especially among older adults seeking treatment [6]. The National Digital Inclusion Alliance defines the digital divide as the gap between those who have affordable access, skills, and support to engage effectively with technology and those who do not, which disproportionately affects people of color, low-income individuals, and older adults [7]. A recent systematic review of older adults’ experiences with digital mental health interventions identified multiple barriers to adoption, including doubts about usefulness, low socioeconomic status, limited access to technology, low levels of digital literacy, and technology anxiety [8]. To address these barriers, a tripartite framework has been proposed that emphasizes access to technology, skills training, and sustained support. Programs such as DOORS (Digital Outreach for Obtaining Resources and Skills), developed at Beth Israel Deaconess Medical Center, exemplify this approach by providing digital devices, digital literacy instruction, and individualized support to promote meaningful engagement with digital mental health interventions [9–12]. While initiatives like DOORS address structural barriers, individual perceptions of usability remain an additional challenge.

Even when access and skills are addressed, the perceived usability of digital health tools remains a critical determinant of sustained use among older adults. Usability, defined as the perceived ease, burden, or intuitiveness of using a digital tool plays a crucial role in adoption, particularly for individuals navigating cognitive, perceptual, or attitudinal barriers [13–17]. Research in usability science has identified several factors influencing the adoption of technology in aging populations, including demographic characteristics (e.g., education, socioeconomic status), psychosocial attributes (e.g., computer anxiety, technology attitudes), and cognitive ability [18–21]. Despite advancements in understanding general technology adoption among older adults, few studies have examined their experiences with emerging digital health tools designed for cognitive and mental health monitoring, particularly smartphone-based digital phenotyping applications [22]. These tools present distinct usability challenges, including privacy concerns, device disruption, and the need for extensive user education, all of which may disproportionately affect older adult populations. To date, few studies have explicitly examined the potential barriers and facilitators to the usability of smartphone-based digital phenotyping tools in this population [23,24].

To address the lack of empirical research in the field, we conducted a secondary analysis of pilot data from a four-week feasibility study conducted at Temple University [23]. The original study assessed the use of a smartphone-based digital phenotyping protocol to evaluate cognition, function, and mood in older adults by using passive GPS data collected from mindLAMP [25], a widely used digital phenotyping app in digital mental health research (see Methods for details). While the original study demonstrated the feasibility of using the app to capture passive digital mobility phenotypes in older adults, it did not examine factors influencing the app’s usability. Thus, this secondary analysis was conducted to explore potential usability-related factors among the same cohort, with the goal of informing future implementation of digital phenotyping tools in aging populations.

To achieve this, we specifically focused on usability outcomes collected during and at the of the four-week study period and examined associations with participant features, including demographics, measures of cognition, attitudes toward computers, mobile device proficiency, and mood. Given prior literature that has shown these factors to be relevant to adoption and usability of other technologies in older adults, we hypothesized that they would relate to usability of the smartphone digital phenotyping application in the present study. While our small sample size (n = 37) limits inferential power and generalizability, these preliminary results are intended to inform future studies on potential usability facilitators and barriers that may influence the adoption of smartphone-based digital phenotyping tools in older adults.

## Methods

### Study procedures

**Fig. 1.**
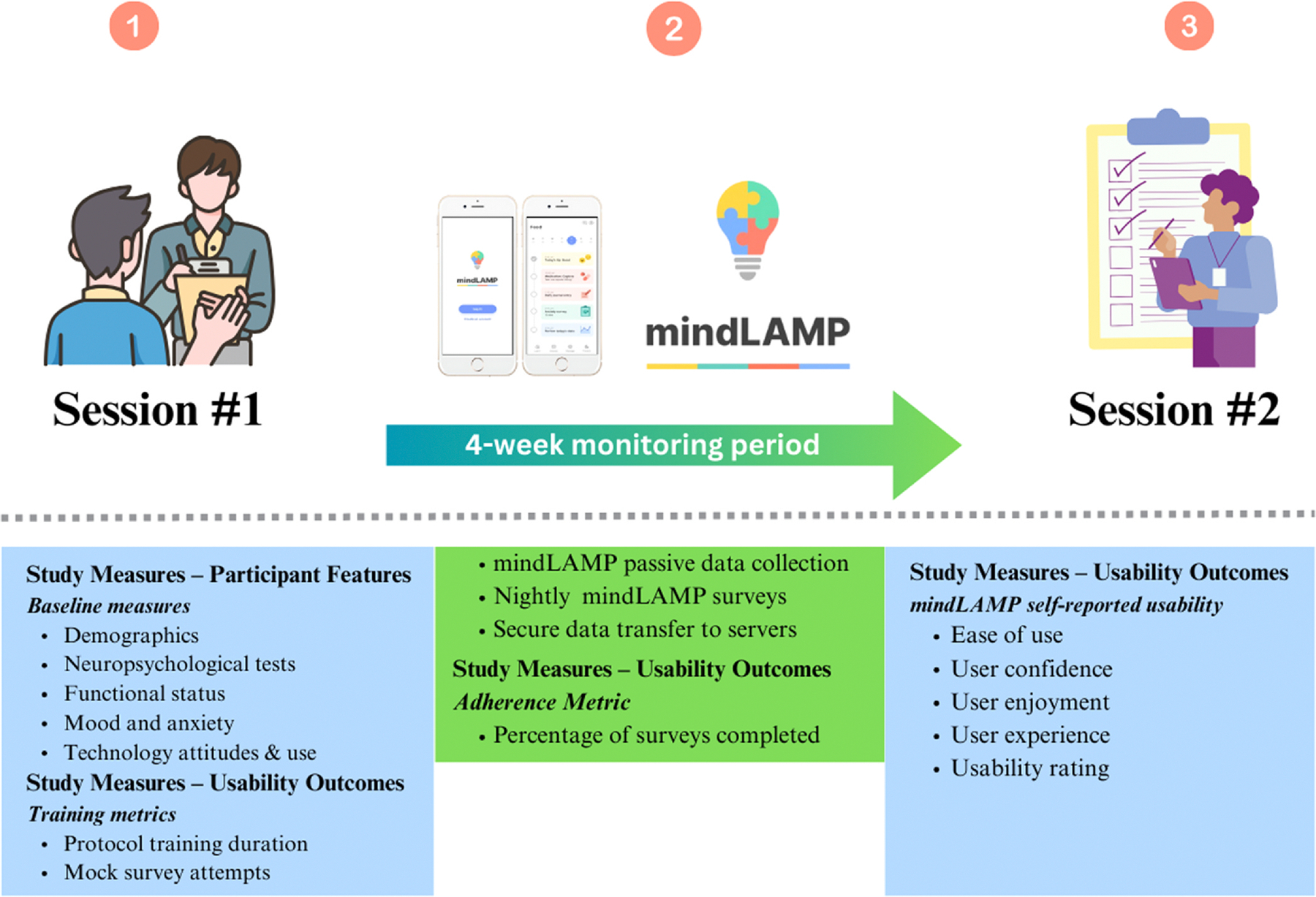
Study overview | This secondary analysis utilized (1) baseline measures from Session 1, including demographics, neuropsychological assessments, attitudes toward computer use, mobile device proficiency, and mood scores. In addition, the protocol training duration and the number of attempts on the mock nightly survey was used as usability outcomes. (2) During the four-week study period, mindLAMP unobtrusively collected passive data and participants completed nightly mindLAMP surveys. The completion percentage of these surveys was used as a usability outcome. (3) At the conclusion of the study, during Session 2, participants responded to debriefing questions regarding their use of mindLAMP throughout the study period.

This study represents a post-hoc analysis of data from a pilot project conducted at Temple University, which assessed the feasibility of a smartphone-based digital phenotyping protocol to evaluate cognition, function, and mood in older adults using passive GPS data gathered via the mindLAMP app. A detailed description of the study methodology can be found in a prior publication [23]. In brief, participants were recruited from specialty dementia clinics and the broader Philadelphia community starting in January 2022 and ending in January 2024. Key eligibility criteria included individuals aged 60 or older with healthy cognition, a diagnosis of mild cognitive impairment (MCI) or mild dementia (Table 1), and ownership of a smartphone (Android or iOS) for at least one year before enrollment. Each participant was also paired with an informant to verify their clinical history and assist with troubleshooting technical issues. Eligible participants were scheduled for an in-person visit (Session 1) and then enrolled in the study for a four-week period. As shown in Fig. 1, participants and their informants attended two study visits, separated by four weeks. At Session 1, participants were briefed on the study procedures, provided informed consent, and had the mindLAMP app configured on their personal smartphones. They then completed a brief training protocol on how to use and interact with the app (see Supplemental Materials 2), followed by a battery of self-report questionnaires and cognitive assessments (Table A2; see Supplemental Materials 1). All neuropsychological assessments were administered by trained research assistants or clinical psychology doctoral students and supervised by a PhD-level board certified neuropsychologist, in a quiet and private room to ensure standard administration conditions. Participants received a US$ 50 payment upon completing the visit. During the four-week study period, mindLAMP passively collected GPS and other sensor data from participants’ smartphones. Participants received a push notification from the app each night to complete a brief five-question survey directly on the app. The questions were designed by the study team and configured on the study app to efficiently capture participants’ mood, subjective cognition, day typicality, percent of day spent away from phone, and any major health/medication changes for each day. At the end of the study period, participants completed a debriefing survey that included questions about their experience using the app, focusing on ease of use, confidence, enjoyment, overall user experience, and usability. This secondary analysis utilized data collected at Session 1 (mindLAMP training metrics, demographics, cognitive test performance, attitudes toward computers, mobile device proficiency, mood), rates of missing survey data during the four-week study period, and self-reported usability ratings from Session 2.

### Study materials – mindLAMP

The mindLAMP app is an open-source digital phenotyping platform developed by the Division of Digital Psychiatry at Beth Israel Deaconess Medical Center for use in both research and clinical settings [25]. It is designed to support self-monitoring of mental health symptoms, delivery of cognitive behavioral therapy (CBT) content, and collect both passive (e.g., GPS, accelerometer) and active (surveys) data for digital phenotyping [26]. The app is freely available on the App Store or Google Play Store and can be installed directly onto participants’ personal smartphones. Once installed and configured, mindLAMP can passively collect sensor data (e.g., GPS, accelerometer) from the device, which is translated into clinically relevant features such as mobility patterns, time spent at home, and step count [27]. The app has four main tabs (Learn, Assess, Manage, and Portal) that allows users to complete mental health surveys, view trends in symptoms over time, engage with self-guided CBT and psychoeducational modules, and access cognitive games [25]. In the original feasibility study, the app was installed on each participant’s smartphone and configured to collect GPS data continuously throughout the four-week study period. Participants were required to open the app once a day to complete a brief five-item evening survey. Participants did not use the app’s other components including the interventional or psychoeducational modules or cognitive games, as these features fell outside the scope of the original study aims.

### Study measures – participant features

#### Neuropsychological Tests.

A battery of tests was administered during Session 1 of the original pilot study [23] to assess various cognitive domains. Global cognition was evaluated using the Mini-Mental State Examination [28]. Attention was measured with the Trail Making Test-Part A [29] and the Wechsler Memory Scale-Revised Digit Span Forward [30]. Processing speed was assessed using the Salthouse Letter-Comparison and Pattern Comparison Test [31]. Executive function was measured with the Trail Making Test-Part B [29] and the Wechsler Memory Scale-Revised Digit Span Backward [30]. Episodic memory was evaluated using the Hopkins Verbal Learning Test-Revised Delayed Recall [32] and the Brief Visuospatial Memory Test-Revised Delayed Recall [33]. Language function was assessed through Animal Fluency [34] and the Boston Naming Test, 30-item version [35]. Raw scores from these assessments were transformed into demographically corrected t-scores [23] using the Calibrated Neuropsychological Normative System. Composite scores for each cognitive domain were calculated by averaging the t-scores for the two tests within each category.

#### Technology Attitudes and Use.

A modified version of the validated 35-item Attitudes Toward Computers Questionnaire was used to assess attitudes related to technology use [36]. In the pilot study, participants completed four of the seven dimensions of the original scale: comfort, efficacy, interest and utility (the dehumanization, control, and gender equality dimensions were removed a priori to reduce participant burden and shorten questionnaire length). The scale had 5-point Likert responses ranging from “strongly agree” to “strongly disagree” to statements such as “Computers make me feel dumb” and “Computers are not too complicated for me to understand”. A composite Global Attitude Towards Computers Score (GATCS) was created by summing across all attitude domains per participant. Higher GATCS scores reflect more positive attitudes towards computers. Computer related anxiety was measured using the Computer Anxiety Subscale [37], which is a 10-item subscale of the Computer Attitudes Scale. Factor-analytic studies and reliability coefficients have indicated that each subscale of the Computer Attitudes Scale is distinct enough to be used separately [37,38]. Responses are on a 4-point Likert scale ranging from “strongly agree” to “strongly disagree” to statements such as “I do not feel threatened when others talk about computers” and “I would feel at ease in a computer class”. Higher scores on the Computer Anxiety Subscale indicate *less* anxiety about computers (i.e., more favorable attitudes towards computers). The Mobile Device Proficiency Questionnaire was administered to participants to measure digital literacy across eight domains: a) Mobile Device Basics, (b) Communication, (c) Data and File Storage, (d) Internet, (e) Calendar, (f) Entertainment, (g) Privacy, and (h) Troubleshooting and Software Management [39]. A Global Mobile Device Proficiency score (GMDPS) was created by summing over all proficiency domains; higher scores indicate better proficiency. Participants completed a 6-item Habitual Smartphone Behavior subscale (5-point scale, α = 0.89) and a 5-item Social Smartphone Use subscale (5-point scale, α = 0.73) [40] to assess smartphone use patterns; higher scores reflect more frequent or habitual smartphone use in general and for communicating with others, respectively. Responses for both subscales ranged from “strongly agree” to “strongly disagree”, to questions such as “Checking my smartphone is becoming a habit” and “I use my smartphone to interact with people”. Participants were also asked to indicate the type of smartphone operating system they used, whether Android or iOS [23].

#### Mood and Anxiety.

Participants completed a 15-item Geriatric Depression Scale [41] to measure depressive symptoms. Participants respond in a “Yes/No” format to questions such as “Are you basically satisfied with your life?” and “Have you dropped many of your activities and interests?”. To assess symptoms of anxiety in our older cohort, the Geriatric Anxiety Inventory [42] was utilized. The Geriatric Anxiety Inventory is 20-item scale with “Agree/Disagree” responses to statements such as “ I often cannot enjoy things because of my worries” and “I think of myself as a nervous person”.

#### Functional Status.

Self- and informant-reported everyday functioning was assessed using the Functional Activities Questionnaire [43]. For participants with healthy cognition, self-reported Functional Activities Questionnaire scores were used, while for those with MCI or dementia, informant-reported Functional Activities Questionnaire scores were utilized.

#### Demographics.

Participants self-reported their sex assigned at birth, race, age, cohabitation status, occupational status, educational attainment, highest household annual income and current marital status.

### Study measures – mindLAMP usability outcomes

#### Training metrics.

After providing informed consent, participants were assisted by study staff to download mindLAMP on their personal smartphones and configure phone settings accordingly, including allowing continuous GPS data capture and enabling push notifications for the nightly survey. Afterwards, participants were guided through a brief training protocol to ensure they would be able to effectively use the app at home during the study period. The training protocol included a PowerPoint presentation with screenshots and other visual aids to review: (1) basic functions of mindLAMP; (2) how to respond to the nightly survey; (3) what to do if you miss a nightly survey; and (4) troubleshooting tips and reminders - including what to do if you are logged out, keep the phone charged, do not enter low battery or airplane mode. Participants completed a guided demonstration of a mock nightly survey to demonstrate their ability to use the mindLAMP survey functionality and interact with the app. The number of attempts of this mock survey was recorded, as was the duration of the entire training protocol. After Session 1 participants were provided a binder including printed slides from the training protocol to facilitate troubleshooting at home (see Supplemental Materials 2 for the full training materials).

#### Adherence.

To measure adherence to the mindLAMP study protocol, which required completion of nightly survey responses over the 4-week study period, we report rates of completed survey data (% of days during which the survey was submitted).

#### Self-reported usability.

Participants’ experience using the study app over the study period was captured at Session 2 using the mindLAMP Use Survey, which is a 5-item scale developed by the Division of Digital Psychiatry at Beth Israel Deaconess Medical Center. Participants responded to questions on a 5-point Likert scale with options ranging from “strongly disagree” to “strongly agree” to questions like “How would you rate your experience with mindLAMP?” and “How would you rate mindLAMP’s usability?”. A composite usability score was created by summing the scores across all questions of the mindLAMP Use Survey. Higher scores indicate more favorable perceptions of the app’s usability.

### Statistical Analysis

All analyses for this post hoc study were conducted using R (version 4.4.1) in RStudio (version 2024.04). In our prior publication [23], which focused on deriving mobility phenotypes from GPS data, three participants were excluded from validation analyses due to major medical events during the study period. For the current analysis, which focuses on participants’ experience using the app, the full cohort (n = 37) was included.

Descriptive statistics, including means, standard deviations, percentages, and score ranges, were used to summarize participant demographics, baseline characteristics (cognition, attitudes toward computer use, mobile device proficiency, and mood; see Table 2), and mindLAMP usability outcomes. Usability outcomes included both self-reported perceptions of use and app training and adherence metrics. There were no missing data for any variables in the feature set.

Exploratory analyses were first conducted to examine the relationships among baseline participant features (e.g., cognition, technology attitudes and use, mood) and demographic variables, prior to analyzing associations with app usability outcomes. Kruskal-Wallis and Mann-Whitney U tests were used to assess differences in continuous baseline features across categorical demographic variables. Spearman correlation analyses were conducted to examine relationships among all continuous baseline measures, including cognition, mood, technology attitudes and mobile device proficiency, age, and years of education.

Our primary analyses focused on examining the associations between app usability outcomes (training metrics, survey adherence, and self-reported usability) and baseline participant characteristics, including demographics, cognition, mobile device proficiency, attitudes toward computers, and mood. The Kruskal–Wallis Test and Mann–Whitney U tests were used to assess usability differences across categorical demographic variables, and Spearman correlation analyses were conducted to examine the associations between usability outcomes and continuous baseline measures.

P-value adjustment using the Benjamini-Hochberg (BH) procedure to control the false discovery rate was planned a priori to address multiple comparisons. Corrections were applied separately to our three sets of analyses: (a) exploratory associations between continuous baseline features (e.g., self-reported technology attitudes and proficiency) and categorical demographic variables (n = 20 tests; Supplemental Figs. A1–A4), (b) exploratory associations among continuous baseline measures (n = 120 tests; Supplemental Table A1), and (c) associations between usability outcomes (including subscores) and participant features, including demographic variables and other baseline measures (n = 100 tests; Table 3 and Supplemental Figs. A5–A8). Following p-value adjustment using the BH procedure, statistical significance was evaluated at an alpha level of 0.05.

## Results

Descriptive statistics for the study cohort’s demographic profile are presented in Table 1, and baseline measures of cognition, attitudes toward computers, mobile device proficiency, and mood are shown in Table 2.

### Training metrics

On average, participants completed the training protocol on how to use the study app in 20.2 min (SD = 6.5) and required only 1.0 attempt (SD = 0.2) to complete the mock nightly survey during Session 1.

### Adherence metrics

As per the study protocol, participants were required to complete a 5-item nightly survey on the mindLAMP app during the 4-week study period. The mean percentage of surveys completed by participants was 80 % (SD = 20 %).

### Self-reported mindLAMP usability

Participants rated their use of mindLAMP favorably, with a mean composite usability score of 19.4 (SD = 4.5) out of a maximum possible score of 25. For specific mindLAMP use domains, ease of use had a mean score of 4.2 (SD = 1.3) out of 5, user confidence 4.2 (SD = 1.2) out of 5, user enjoyment 3.4 (SD = 1.1) out of 5, overall experience 3.7 (SD = 1.0) out of 5, and usability rating 4.1 (SD = 1.0) out of 5.

### Relations among participant baseline measures

Differences in baseline self-reported measures, including attitudes toward computers, mobile device proficiency, computer anxiety, and habitual and social smartphone use, were examined across categorical demographic variables such as sex, race, education, and phone type, as shown in Supplemental Figs. A1–A4; see Supplemental Materials 1. Although male participants initially reported higher habitual smartphone use (*P* = .02, *P*_*adjusted*_ =.15), and social smartphone use (*P* = .009, *P*_*adjusted*_ =.15) compared to female participants, these differences did not remain statistically significant after correcting for multiple comparisons. No significant differences in baseline measures were observed across sex, race, education, or phone type before or after correcting for multiple comparisons.

Associations among continuous participant variables, including age, years of education, cognition, mood, attitudes towards computers, mobile device proficiency and habitual and social smartphone use, are presented in Supplemental Table A1; see Supplemental Materials 1. After correction for multiple comparisons, the association between attitudes toward computers and computer anxiety remained statistically significant, indicating that participants with more positive attitudes toward computers also reported lower levels of computer anxiety (*r* = .73, *P*_*adjusted*_ < .001). Several other correlations between education, cognitive scores, computer attitudes, mobile device proficiency, and geriatric anxiety reached nominal significance but did not remain significant after correcting for multiple comparisons.

### Relations between App usability and participant features

Differences in usability outcomes across sex, race, education, and phone type are presented in Supplemental Figs. A5 to A8 (see Supplemental Materials 1). While female participants initially appeared to complete the training protocol faster than male participants (*P* = .02), this difference did not remain statistically significant after correcting for multiple comparisons (*P*_*adjusted*_ =.46). Similarly, Black participants reported higher scores compared to participants of other races for app enjoyment (*P* = .04, *P*_*adjusted*_ =.46) and perceived usefulness (*P* = .04, *P*_*adjusted*_ =.46), however, these differences did not remain significant after correction. A comparable pattern was observed for education, with participants who had completed some college reported higher composite usability scores (*P* = .01, *P*_*adjusted*_ =.46) than those with either a high school education or a bachelor’s degree or higher, along with higher scores perceived usefulness of mindLAMP (*P* = .02, *P*_*adjusted*_ =.46), though these too did not survive multiple comparison correction. No statistically significant differences emerged for usability outcomes across phone type. Associations between usability outcomes and participant age, education, cognition, mood, attitudes toward computers, and mobile device proficiency are presented in Table 3. Although associations such as between education and composite usability yielded a *P*-value less than 5 %, none remained statistically significant after correcting for multiple comparisons.

## Discussion

As the population of older adults facing complex medical, mental health, and cognitive challenges continues to rise, there is a growing need for effective, efficient, and personalized intervention strategies. Digital health tools offer a promising avenue to address these unmet needs, however, their successful adoption depends on older adults’ ability to engage with these tools. In this post hoc analysis, we evaluated both objective and subjective usability of mindLAMP, a smartphone-based digital health app capable of capturing both active survey and passive sensor data from personal smartphone devices. Building on prior work in digital technology adoption among older adults, we examined demographic, cognitive, attitudinal, and technological factors associated with usability outcomes. While preliminary, our findings suggest that smartphone-based digital mental health interventions incorporating digital phenotyping approaches may be usable for subsets of older adults who receive modest support and training. These results also highlight potential candidate factors that may inform future implementation efforts, though our findings should be interpreted with cautious given the small, highly educated and technologically proficient sample.

On average, participants required approximately 20 min to complete the study app training protocol and needed only a single attempt to successfully complete the practice daily surveys. Over the 4-week study period, participants completed an average of 80 % of daily surveys, reflecting high adherence. Self-reported perceptions of usability collected at the end of the study indicated generally favorable impressions, with a mean composite usability score of 19.4 out of 25. Most participants (81.1 %) agreed or strongly agreed that mindLAMP was easy to use, 86.5 % expressed confidence in using the app, and 70.2 % reported a positive overall experience. Only 10.8 % reported experiencing any difficulty. These findings suggest that with modest initial training, older adults may be able to successfully engage with smartphone-based digital phenotyping apps for health-related survey completion over an extended period. Although promising, our cohort of older adults at baseline were highly educated and technologically competent, which may have impacted usability positively. Building on these preliminary results, we further explored factors that may influence perceived usability and the broader adoption of digital health tools in older adults.

Analysis of participants’ baseline characteristics revealed a predominantly female cohort with high levels of education and income, the majority of whom met the diagnostic criteria for healthy cognitive status. These factors are known to facilitate the adoption of digital mental health tools among older adults [8]. Neither attitudes toward computers, mobile device proficiency, nor computer anxiety, were significantly associated with use of the app, as measured by survey completion rates during the 4-week study period. Although unexpected based on prior literature [39], similar null results have been reported in other work using smartphone intervention apps with older adults, including the SmartPrompt study which also found no significant associations between technology attitudes or computer proficiency and usability or efficacy outcomes [44]. Nonetheless, these findings should be interpreted with caution, as a possible explanation for our null findings is that our study was underpowered to detect such an association due to our small sample size. Future work should investigate whether app features or specific contexts might mitigate the possible influence of negative attitudes, computer anxiety, and low smartphone proficiency on usability and adherence to digital health tools.

Similarly, though no significant differences in usability outcomes were observed between iPhone and Android users, this finding should also be interpreted with caution given the small and unequal group sizes. Because of the numerous technical and other sociodemographic differences between individuals who own different phone types (i.e., iOS vs. Android) [45–47], it is critical to evaluate the user-experience of digital health tools in larger more balanced samples to ensure equivalence across device types in terms of usability and acceptability.

Although not significant after correction for multiple comparisons, two participant characteristics, education and race, show great promise for understanding usability outcomes in older adults, though these associations differed from patterns reported in the existing literature. Contrary to prior findings that individuals with higher levels of education are more likely to engage with technology and adopt digital mental health interventions [48], our analysis showed that participants with lower educational attainment (high school or some college) reported higher composite usability scores compared to those with at least a bachelor’s degree (Supplemental Figs. A7). This difference appeared to be driven primarily by more favorable ratings on app enjoyment and perceived usefulness (Supplemental Fig. A7). Although the negative correlation between years of education and composite usability followed the same trend, it also did not remain significant after correction for multiple comparisons (Table 3). It is possible that participants with higher education levels may have more critical attitudes toward digital tools due to greater expectations or higher levels of technology-related anxiety. Alternatively, the small number of participants with lower educational attainment in our highly educated sample may represent a particularly motivated subgroup with greater interest in engaging with technology. As such, these findings should be interpreted with caution and warrant further investigation in more diverse and representative samples. We also observed potential race-related differences in usability ratings, as participants who identified as Black reported higher usability scores than White or Asian participants, seen in higher ratings of enjoyment, and perceived usability (Supplemental Fig. A6).

Although not a primary focus of this study, we observed meaningful associations among measures of technology-related beliefs, mobile device proficiency, and computer anxiety. Specifically, relationships among scores on the Attitudes Toward Computers Questionnaire, the Computer Anxiety Scale, and the Mobile Device Proficiency Questionnaire align with prior research demonstrating links between computer attitudes and anxiety [36]. These patterns suggest that these measures may reflect a shared underlying construct related to technology beliefs and experiences, which could be explored as a unified factor in future research.

### Strengths and limitations

This study has several strengths worth noting. First, we incorporated multiple operationalizations of usability, including both subjective (self-reported usability ratings) and objective measures (training time, practice performance, and adherence to daily surveys). Unlike many prior studies that assess usability after a single interaction, participants’ ratings were collected following an extended 4-week period of independent use, allowing for a more ecologically valid assessment of engagement. Additionally, our cohort was extensively characterized at baseline through a comprehensive battery of clinical neuropsychological assessments and validated questionnaires, enabling robust exploration of potential facilitators and barriers to the usability of digital mental health interventions.

However, several important limitations warrant consideration. This was a secondary, post-hoc analysis conducted in a relatively small and demographically homogenous sample. Participants were predominantly highly educated, proficient smartphone users, and held positive attitudes toward technology use. Additionally, the sample was predominantly cognitively healthy, with only one participant diagnosed with dementia (likely due to inclusion criteria requiring independent smartphone use and partially due to difficulties recruiting older adults for in-person research during the COVID-19 pandemic). These factors likely facilitated high usability and adherence, but limit the generalizability of our findings to broader aging populations. Moreover, the small sample size and homogeneity may have reduced statistical power to detect meaningful associations between participant characteristics and app usability outcomes. The study also involved a large number of comparisons relative to the sample size, which increases the risk of type I error despite the application of false discovery rate correction procedures. Furthermore, the usability assessment employed in this study was not a validated scale, which may limit the precision and reliability of participants’ self-reported experiences. As such, null findings should be interpreted with caution, and replication in larger, more diverse samples will be essential to confirm these preliminary results. Finally, our study focused on the usability of a highly specific smartphone digital phenotyping app (mindLAMP). While our findings provide insight into similar smartphone-based digital health tools, they may not generalize to other digital formats such as web-based platforms, wearables, or virtual assistants, which may present distinct usability challenges for older adults. Future research should explore how older adults engage with these alternative technologies to inform inclusive and adaptable digital mental health solutions.

## Conclusion

Overall, our findings suggest that smartphone-based digital mental health tools, including digital phenotyping apps, may be usable for subsets of older adults when supported training is provided. In this pilot study, older adults were able to successfully complete training, adhere to daily survey tasks, and report favorable usability experiences with a smartphone-based digital phenotyping app. These results are preliminary and should be interpreted with caution given the small sample size, the high proportion of participants with a bachelor’s degree or higher, and the exploratory nature of the study. Future development of digital mental health tools should involve older adults directly in the design process, and optimize usability across individuals with diverse educational backgrounds, technology experience, and expectations for digital tools.

## Supplementary Material

Figures

Supplementary Material

## Figures and Tables

**Table 1:** Participant demographic characteristics.

Characteristic	Mean/n	SD/%

Age (years)	72	5.5
Education (years)	16	2.6
**Sex**		
Female	25	67.6 %
Male	12	32.4 %
**Race**		
Asian	2	5.4 %
Black/African American	12	32.4 %
White	22	59.5 %
Not reported	1	2.7 %
**Marital Status**		
Married	22	59.5 %
Widowed	7	18.9 %
Divorced	5	13.5 %
Separated	3	8.1 %
**Education**		
High school	4	10.8 %
Some college	5	13.5 %
≥ Bachelor’s	28	75.7 %
**Occupation Status**		
Full-time	3	8.1 %
Part-time	4	10.8 %
Retired	30	81.1 %
**Highest Annual Income**		
Less than 50,000	6	16.2 %
50,000 to 100,000	12	32.4 %
Greater than 100,000	16	43.3 %
Prefer not to answer	3	8.1 %
**Living Status**		
Live alone	13	35.1 %
Live with others	24	64.9 %
**Phone Type**		
Android	10	27.0 %
iPhone	27	73.0 %
**Cognition Diagnosis**		
Healthy cognition	30	81.1 %
MCI	5	13.5 %
Impaired, not MCI	1	2.7 %
Mild dementia	1	2.7 %

Note. MCI, mild cognitive impairment

**Table 2:** Participant baseline measures.

Baseline measures	Mean	SD	Minimum – Maximum

**Neuropsychological Test (t-score)**			
Global Cognition (MMSE)	52.4	7.8	37 –67
Attention	51.6	6.3	38 –66
Processing Speed	55.0	8.0	36 –74
Executive Function	51.0	7.2	38 –66
Memory	48.1	11.3	20 –75
Language	50.0	9.0	28 –72
**Functional Abilities**			
Functional Activities Questionnaire	1.7	2.4	0 –6
**Computer Attitudes**			
Comfort	20.1	4.1	5 –25
Efficacy	21.7	2.9	5 –25
Interest	21.6	2.7	5 –25
Utility	21.4	2.6	5 –25
GATCS	84.7	10.2	20 –100
**Device Anxiety & Behavior**			
Computer Anxiety	35.7	5.3	10 –40
Social Smartphone Use	8.4	3.0	5 –25
Habitual Smartphone Behavior	9.7	4.3	6 –30
**Mobile Device Proficiency**			
Mobile Device Basics	4.8	0.6	1 –5
Communication	4.8	0.6	1 –5
Data and File Storage	3.6	1.4	1 –5
Internet	4.7	0.8	1 –5
Calendar	4.1	1.4	1 –5
Entertainment	4.3	1.1	1 –5
Privacy	4.1	1.0	1 –5
Troubleshooting and Software Management	4.1	1.3	1 –5
GMDPS	34.4	6.0	8 –40
**Clinical Mood Scores**			
Geriatric Depression	1.6	1.4	0 –5
Geriatric Anxiety	1.6	2.5	0 –20

Note. Neuropsychological test scores are presented as demographically corrected t-scores, derived using the Calibrated Neuropsychological Normative System. Raw scores were adjusted for age, sex, education, and estimated premorbid IQ (assessed via the Hopkins Reading Test).

**Abbreviations:** MMSE, Mini-Mental Status Exam; GATCS, Global Attitude Towards Computers Score; GMDPS, Global Mobile Device Proficiency Score.

**Table 3:** Spearman Correlation Coefficients Between Participant Features and mindLAMP Usability.

	Usability outcomes			

Participant features	GMUI	Training duration	Survey attempts	Surveys completed

Age (years)	−.12	.26	.20	.01
Education	−.37*	.02	.12	.07
Global Cognition (MMSE)	.12	−.04	.13	.29
Attention	.01	−.05	−.12	−.22
Processing Speed	−.10	−.07	−.22	.12
Executive Function	.01	−.24	−.03	.20
Memory	−.15	.27	.28	.06
Language	.15	.20	.27	−.09
Functional Activities Questionnaire	.04	−.04	.27	−.13
GATCS	.24	−.05	−.09	.06
GMDPS	.24	−.25	−.25	−.12
Computer Anxiety	.26	−.09	−.21	−.06
Geriatric Depression	< .01	−.05	−.04	−.03
Geriatric Anxiety	.09	−.08	.26	−.02
Social Smartphone Usage	−.21	.06	−.23	−.07
Habitual Smartphone Behavior	−.35	.43	−.23	−.10

**Abbreviations:** GMUI, Global App Use Index; MMSE, Mini-Mental Status Exam; FAQ, Functional Activities Questionnaire; GATCS, Global Attitude Towards Computers Score; GMDPS: Global Mobile Device Proficiency Score.

No correlation coefficients remained significant after Benjamini-Hochberg correction (q < .05)

* P < .05 (2-tailed)

## Data Availability

The non-identifiable datasets analyzed during this post-hoc study are available on request from author(s) Tania Giovannetti and Katherine Hackett with an appropriate IRB and data sharing agreement.

## Appendix A. Supporting information

Supplementary data associated with this article can be found in the online version at doi:10.1016/j.inpsyc.2025.100123.
