# Supplementary material for: Barriers and facilitators to usability of a smartphone-based digital mental health tool in older adults: Insights from a secondary analysis of mindLAMP
Source: Int Psychogeriatr. Author manuscript; Available in PMC 2026 May 5. (PMC13142898; doi:10.1016/j.inpsyc.2025.100123)
Supplement: Figures [file NIHMS2162122-supplement-Figures.docx]

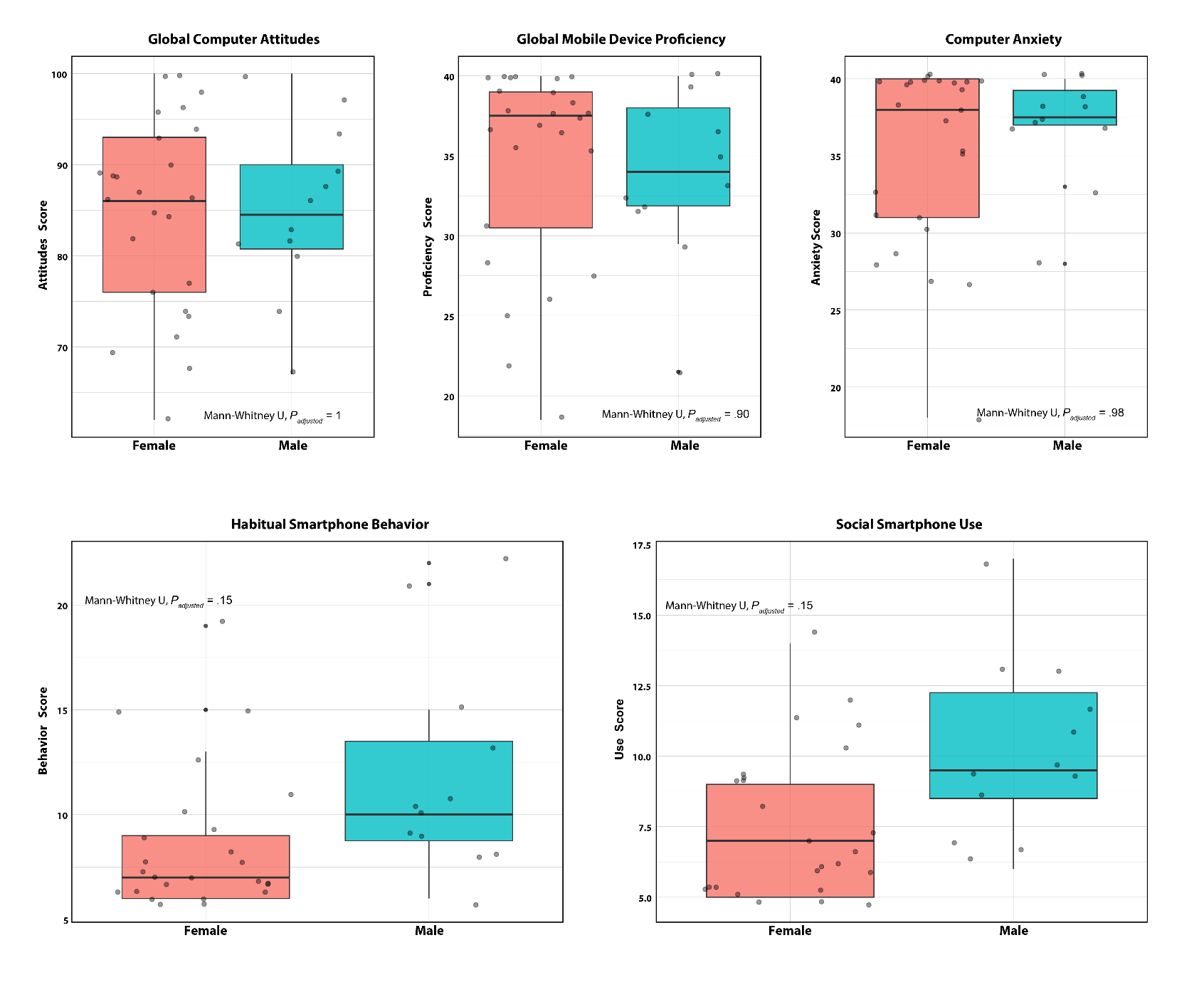


**Figure 1** Boxplots illustrating the distribution of participant baseline measures across Sex


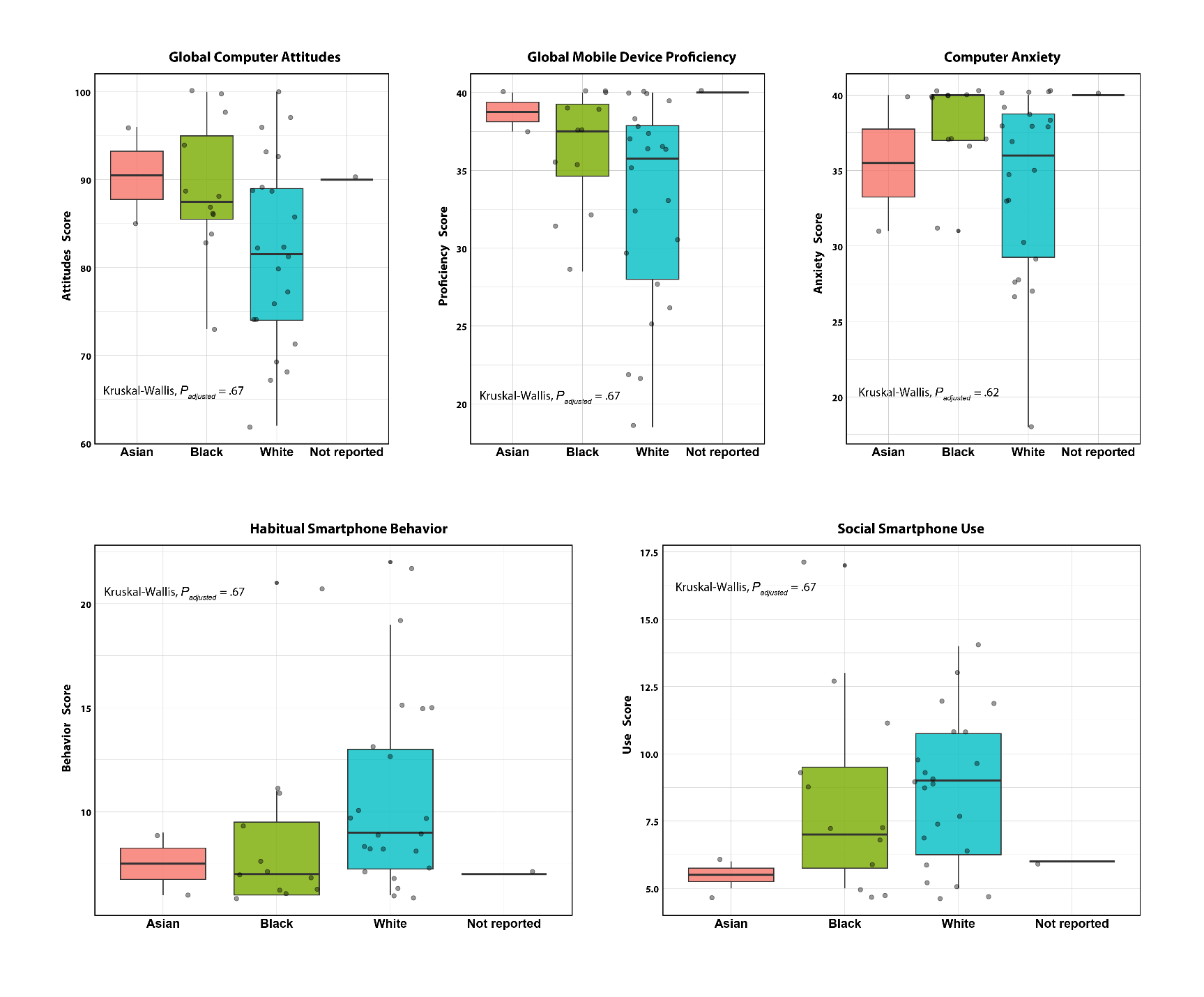


***Figure A2.*** *Boxplots illustrating the distribution of participant baseline measures across racial groups*


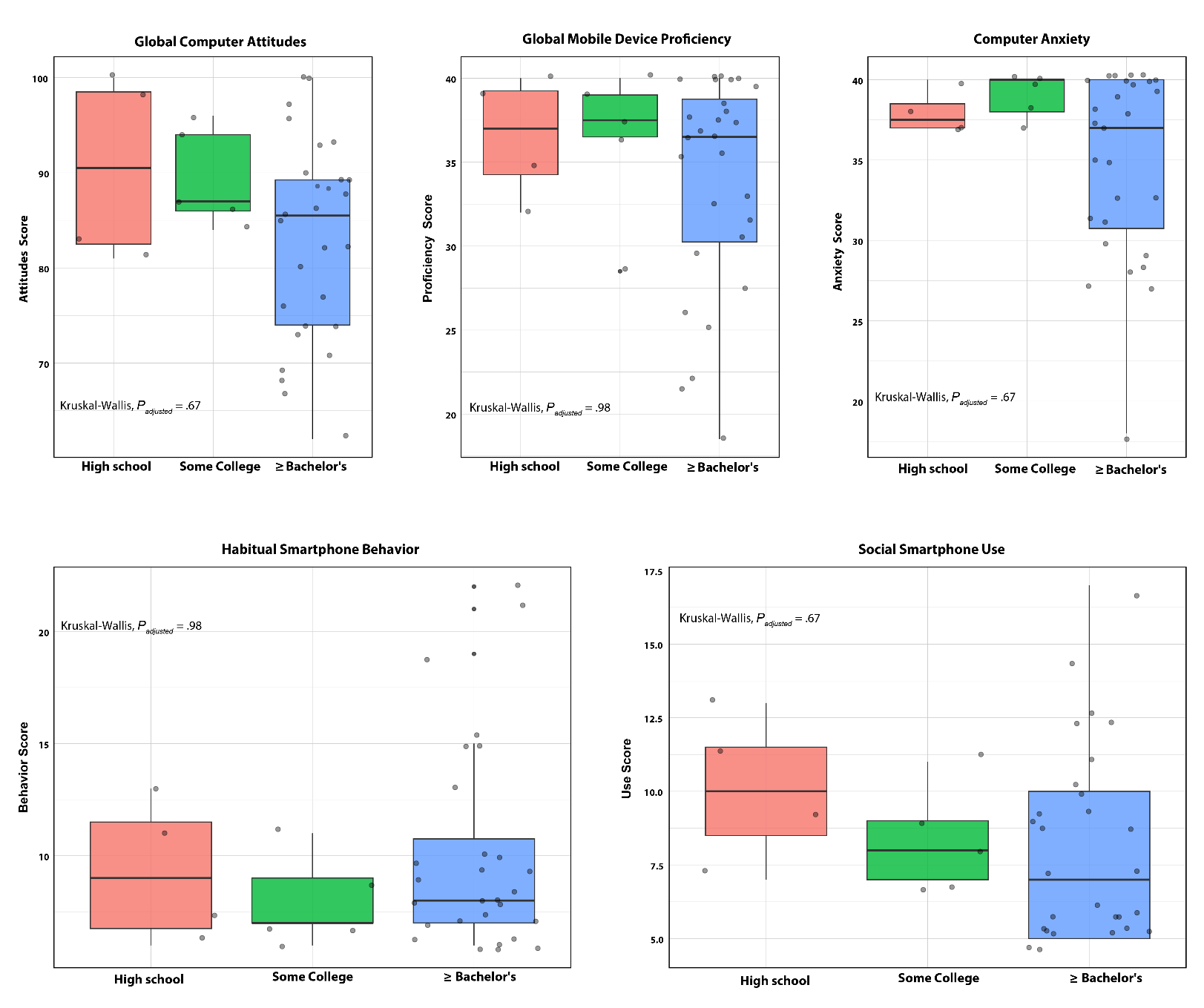


**Figure A3.** Boxplots illustrating the distribution of participant baseline measures across educational levels


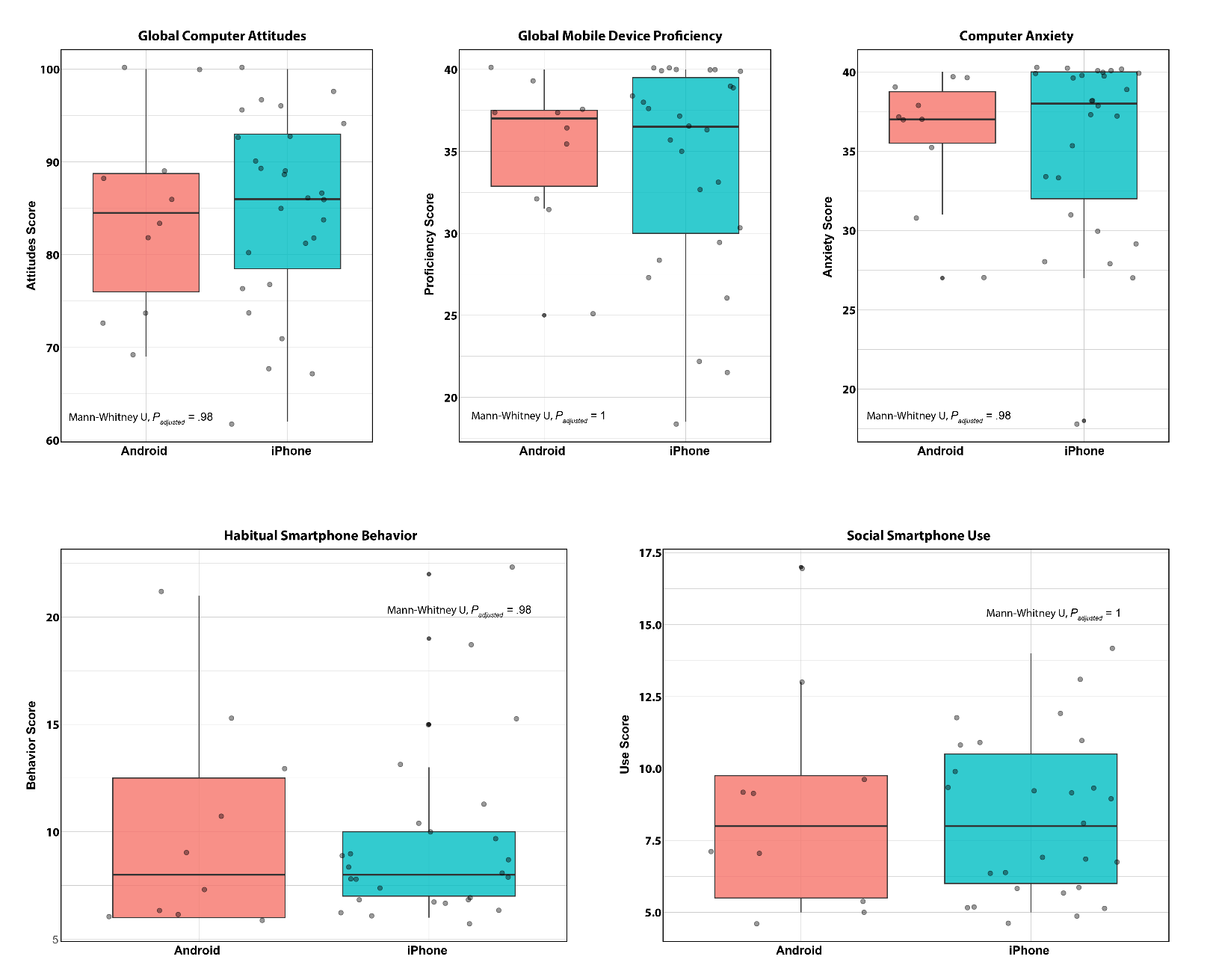


***Figure A4.*** *Boxplots illustrating the distribution of participant baseline measures across phone types*


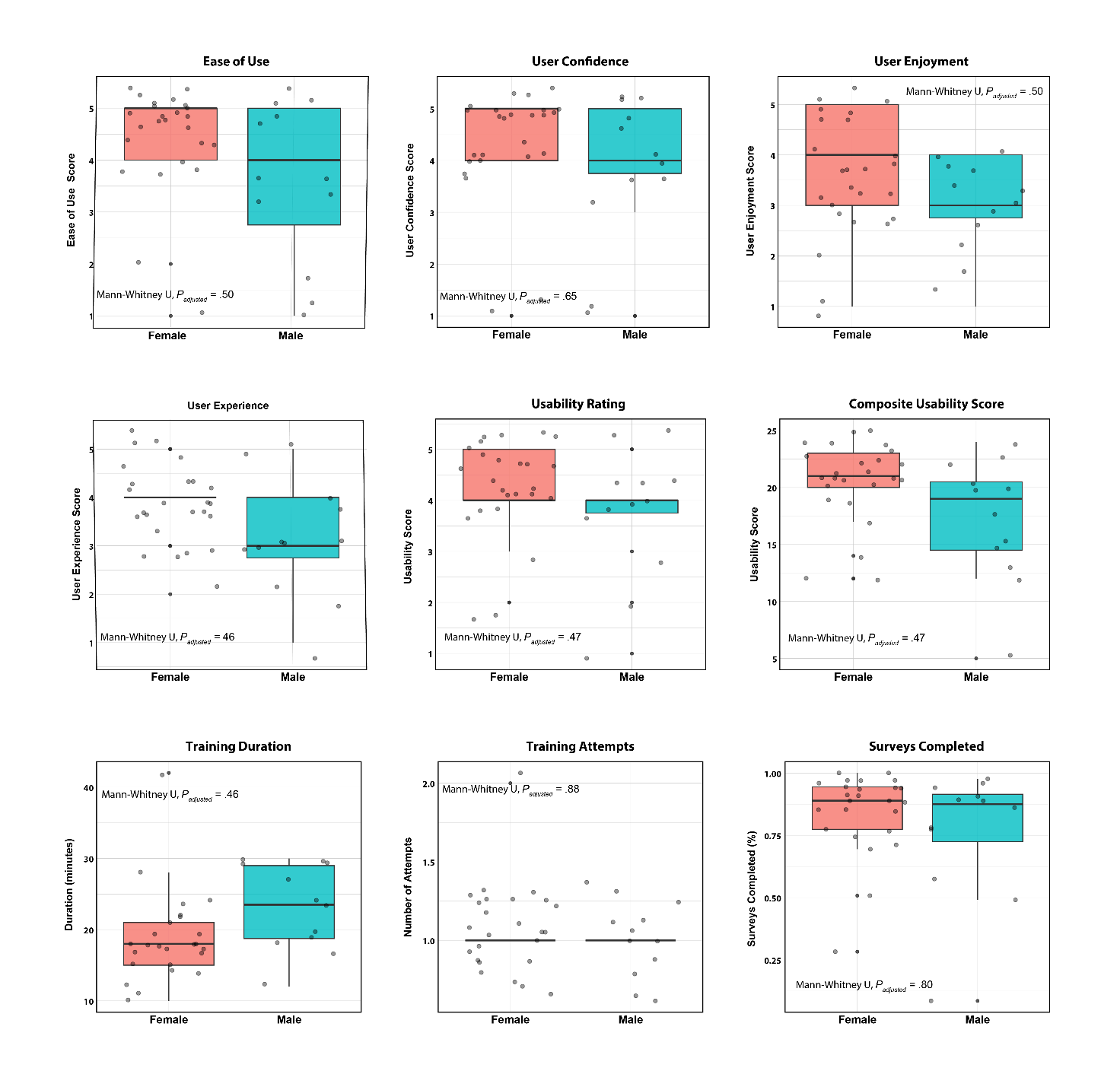


***Figure A5****. Boxplots illustrating the distribution of Study App Usability Outcomes across Sex*


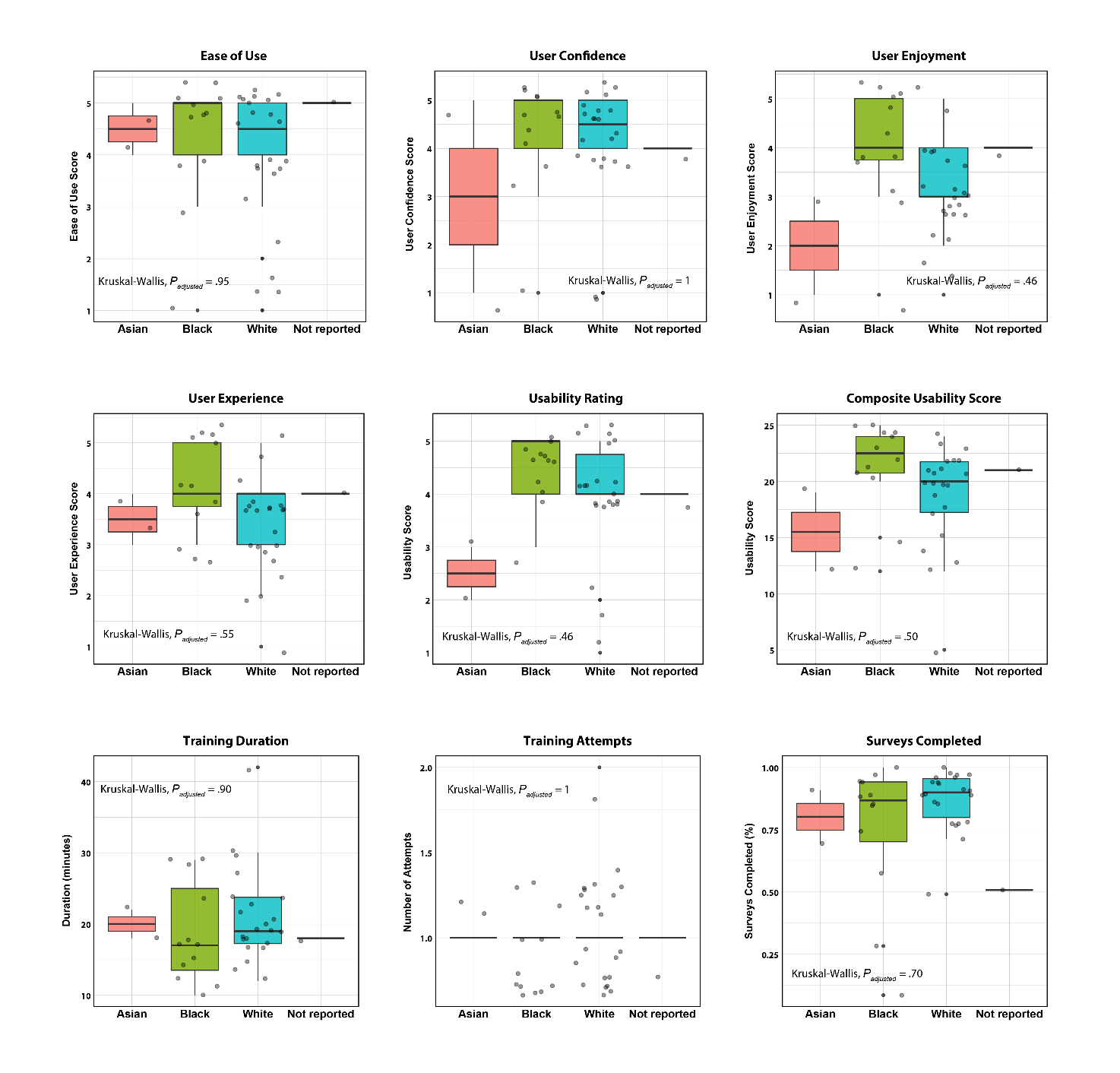


***Figure A6****. Boxplots illustrating the distribution of Study App Usability Outcomes across Race*


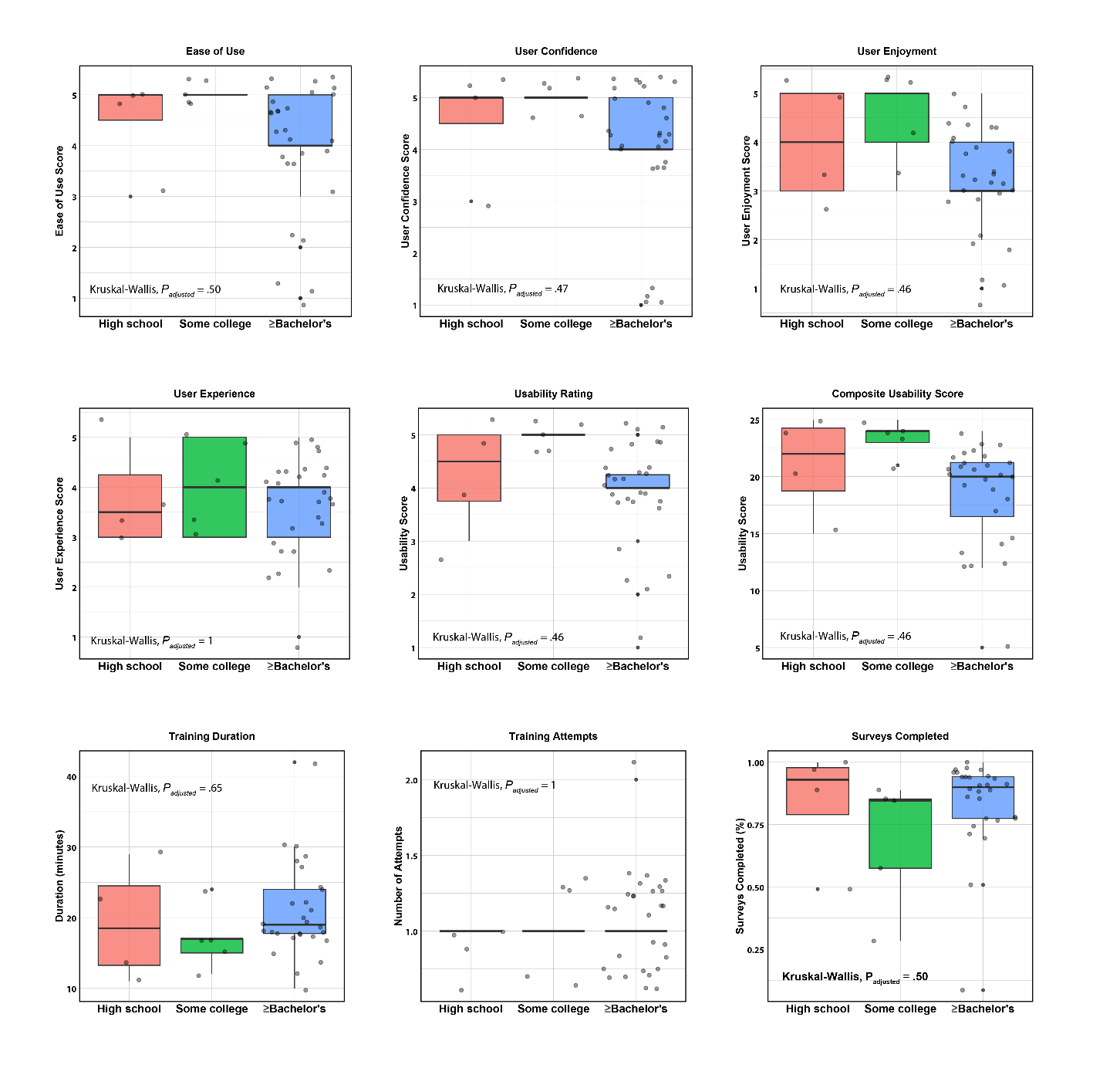


***Figure A7****. Boxplots illustrating the distribution of Study App Usability Outcomes across levels of Education*


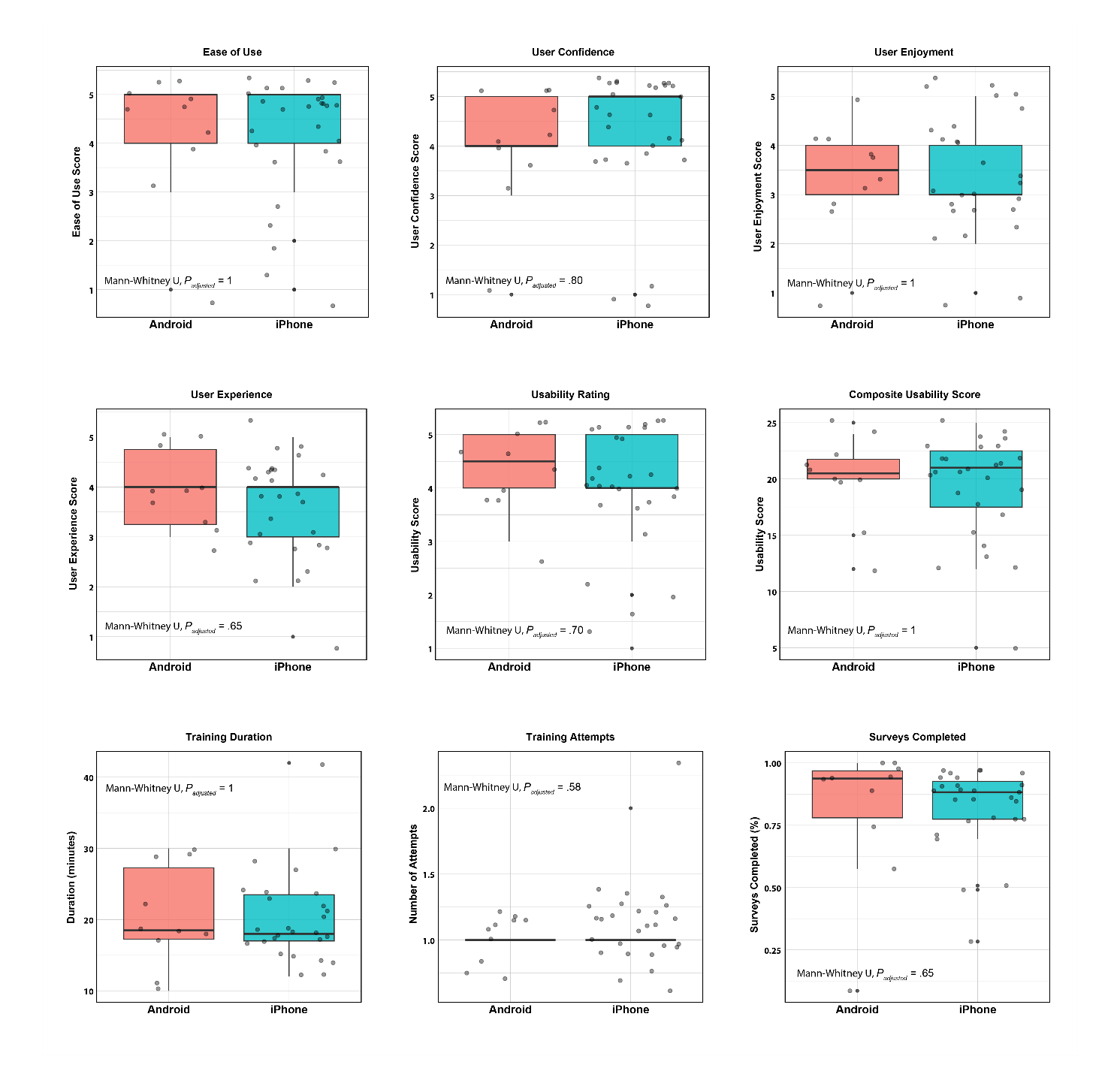


***Figure A8****. Boxplots illustrating the distribution of Study App Usability Outcomes across Phone Type*

**Table A1.** Spearman Correlations Among Baseline Cohort Measures of Interest

| **Variable** | **1** | **2** | **3** | **4** | **5** | **6** | **7** | **8** | **9** | **10** | **11** | **12** | **13** | **14** | **15** | **16** |
| --- | --- | --- | --- | --- | --- | --- | --- | --- | --- | --- | --- | --- | --- | --- | --- | --- |
| 1. Age |  |  |  |  |  |  |  |  |  |  |  |  |  |  |  |  |
| 1. Education | -.02 |  |  |  |  |  |  |  |  |  |  |  |  |  |  |  |
| 1. Global Cognition (MMSE)^a^ | -.27 | -.12 |  |  |  |  |  |  |  |  |  |  |  |  |  |  |
| 1. Attention | .37^d^ | .14 | -.17 |  |  |  |  |  |  |  |  |  |  |  |  |  |
| 1. Processing Speed | .08 | .10 | -.10 | .33^d^ |  |  |  |  |  |  |  |  |  |  |  |  |
| 1. Executive Function | .28 | .27 | .14 | .43^e^ | .41^d^ |  |  |  |  |  |  |  |  |  |  |  |
| 1. Memory | -.14 | .07 | .26 | -.29 | -.14 | -.04 |  |  |  |  |  |  |  |  |  |  |
| 1. Language | .35^d^ | -.23 | .06 | .10 | .13 | .02 | -.13 |  |  |  |  |  |  |  |  |  |
| 1. GATCS^b^ | -.09 | -.33^d^ | .28 | -.01 | .06 | -.08 | -.08 | .28 |  |  |  |  |  |  |  |  |
| 1. GMDPS^c^ | -.30 | -.31 | .13 | -.04 | .22 | -.12 | -.16 | .15 | .47^e^ |  |  |  |  |  |  |  |
| 1. Geriatric Depression | -.01 | -.03 | -.15 | .01 | .32 | .15 | .24 | -.05 | -.11 | -.13 |  |  |  |  |  |  |
| 1. Geriatric Anxiety | -.01 | -.10 | -.04 | .02 | .17 | .05 | .03 | .01 | -.36^d^ | -.15 | .45^e^ |  |  |  |  |  |
| 1. Computer Anxiety | -.09 | -.32 | .13 | .02 | -.02 | -.11 | -.15 | .27 | .73^e,f^ | .48^e^ | -.01 | -.40^d^ |  |  |  |  |
| 1. Functional Activities Questionnaire | .12 | .08 | -.08 | -.12 | .07 | .12 | -.05 | .27 | -.23 | -.16 | .45^e^ | .53^e,f^ | -.17 |  |  |  |
| 1. Social Smartphone Usage | .20 | -.16 | .05 | .18 | -.07 | -.04 | .04 | -.11 | -.13 | -.24 | .09 | -.01 | -.26 | <.01 |  |  |
| 1. Habitual Smartphone Behavior | .21 | -.03 | -.14 | .11 | -.06 | -.09 | .19 | -.15 | -.21 | **-**.31 | .06 | .03 | -.30 | .01 | .75^e,f^ |  |

Note: MMSE, Mini-Mental Status Exam; GATCS, Global Attitude Towards Computers Score; GMDPS, Global Mobile Device Proficiency Score. Benjamini-Hochberg correction was applied to *P*-values from 120 hypothesis tests.

^d^ P<.05 (2-tailed)

^e^ P<.01 (2-tailed)

^f^ depicts correlation coefficients that remain significant after Benjamini-Hochberg correction

**Table A2.** Overview of Study Assessments by Domain and Session

| **Assessment** | **Domain** | **Session** |
| --- | --- | --- |
| **Neuropsychological Tests** | | |
| Mini-Mental State Examination | Global Cognition | Session 1 |
| Trail Making Test-Part A | Attention | Session 1 |
| Wechsler Memory Scale-Revised Digit Span Forward | Attention | Session 1 |
| Salthouse Letter-Comparison Test | Processing Speed | Session 1 |
| Pattern Comparison Processing Speed Test | Processing Speed | Session 1 |
| Trail Making Test-Part B | Executive Function | Session 1 |
| Wechsler Memory Scale-Revised Digit Span Backward | Executive Function | Session 1 |
| Hopkins Verbal Learning Test-Revised Delayed Recall | Episodic Memory | Session 1 |
| Brief Visuospatial Memory Test-Revised Delayed Recall | Episodic Memory | Session 1 |
| Animal Fluency | Language Function | Session 1 |
| Boston Naming Test | Language Function | Session 1 |
| **Functional Ability** | | |
| Functional Activities Questionnaire | Functional Status | Session 1 |
| **Mood** |  |  |
| Computer Anxiety Subscale | Mood |  |
| Geriatric Depression Scale | Mood | Session 1 |
| Geriatric Anxiety Inventory | Mood | Session 1 |
| **Attitudes & Use** | | |
| Attitudes Toward Computers Questionnaire | Technology Attitudes | Session 1 |
| Mobile Device Proficiency Questionnaire | Technology Use | Session 1 |
| **Behavioral Assessments** | | |
| Habitual Smartphone Behavior subscale | Technology Use | Session 1 |
| Social Smartphone Use subscale | Technology Use | Session 1 |
| **mindLAMP usability-Training** | | |
| Protocol Training Duration | Usability – Training | Session 1 |
| Mock Survey Attempt Count | Usability – Training | Session 1 |
| Percentage of Surveys Completed | Usability – Adherence | 4-week Study Period |
| **mindLAMP usability-Perception** | | |
| Ease of Use | Usability – Self-Report | Session 2 |
| User Confidence | Usability – Self-Report | Session 2 |
| User Enjoyment | Usability – Self-Report | Session 2 |
| User Experience | Usability – Self-Report | Session 2 |
| Usability Rating | Usability – Self-Report | Session 2 |
