# Supplementary material for: Barriers and facilitators to usability of a smartphone-based digital mental health tool in older adults: Insights from a secondary analysis of mindLAMP
Source: Int Psychogeriatr. Author manuscript; Available in PMC 2026 May 5. (PMC13142898; doi:10.1016/j.inpsyc.2025.100123)

Using the mindLAMP App -- *iPhones*

Participant & Informant Training Procedures

Examiner: record start time
(& whether in-person or via zoom)

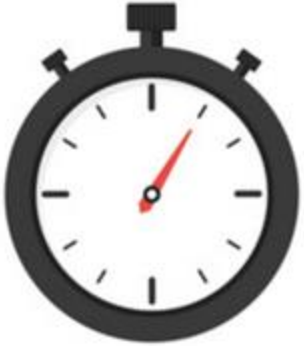

Basic Functions

The mindLAMP application is designed to:

1. Collect de-identified data from your phone sensors throughout the day without any interference while you go about your regular daily activities.

2. Send you a notification each night to complete a brief 5-question survey on your phone. The survey should take about **3 minutes** to complete.

This is what the app will look like on your phone screen.

(show on participant's phone)

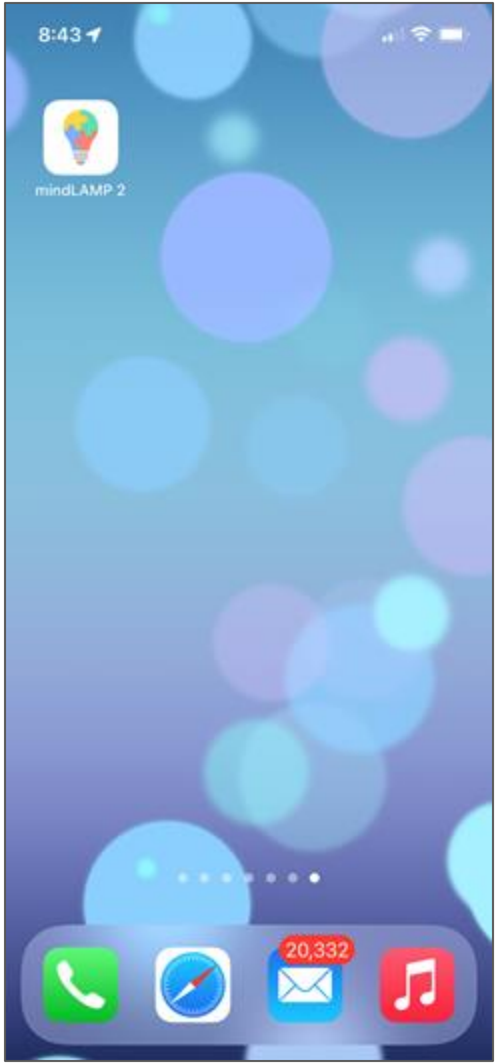

Responding to the nightly survey

In the next few slides, we will show you how to respond to the nightly survey on your phone!

You will receive a notification to complete the survey at the same time, each night, for the next 4 weeks.

Your nightly survey notification is scheduled for ________ pm

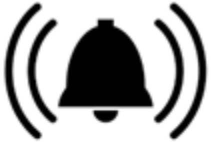

STEP 1:
Survey notification will appear

You will hear or see a notification on your phone...

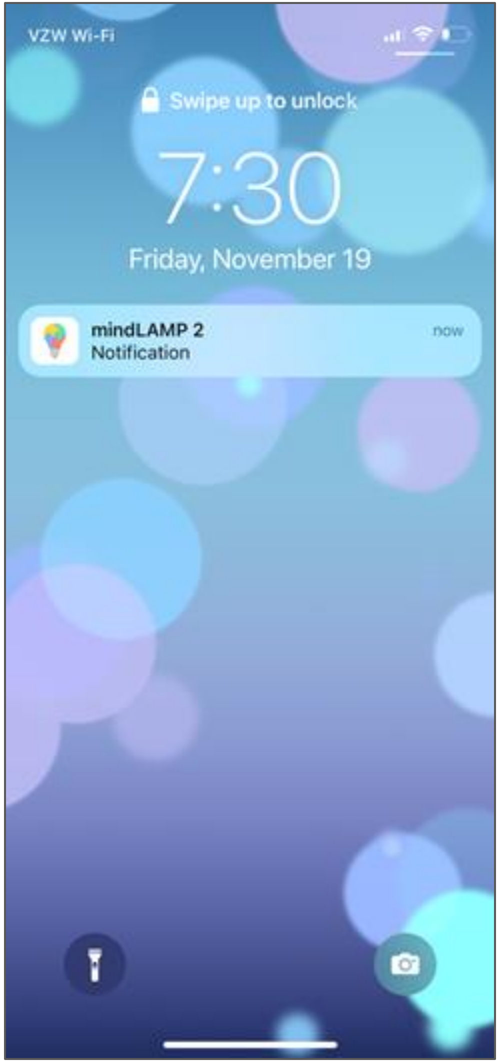

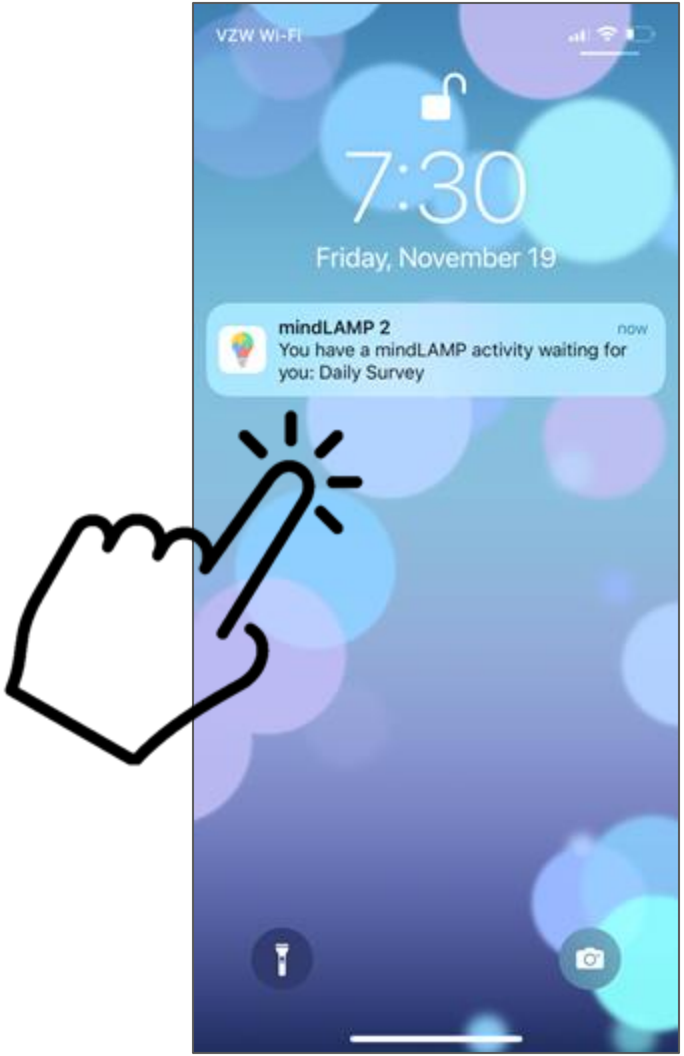

Responding to the survey notification

If your phone is locked, you'll need to unlock your phone (using your passcode, touch id, or face id) after tapping the notification

If you are using your phone when you get an alert, the notification will look like this...

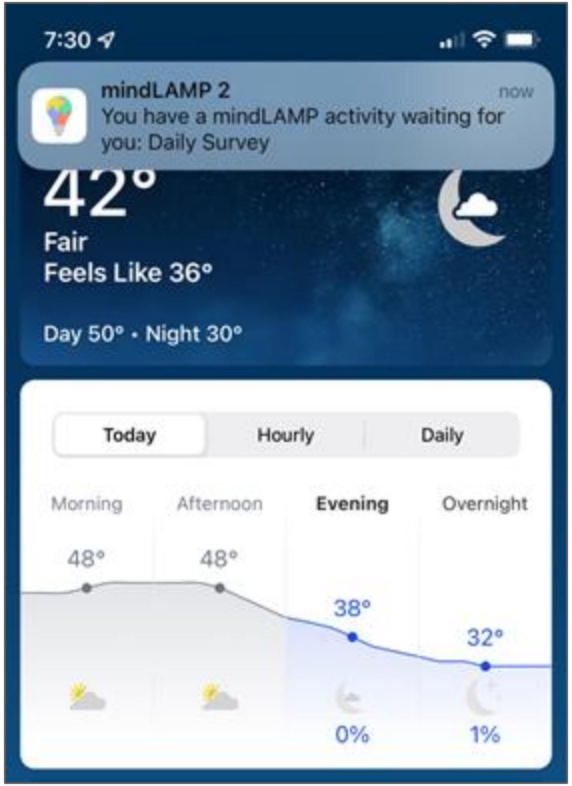

When you hear the alarm and see the notification...

Tap on the notification

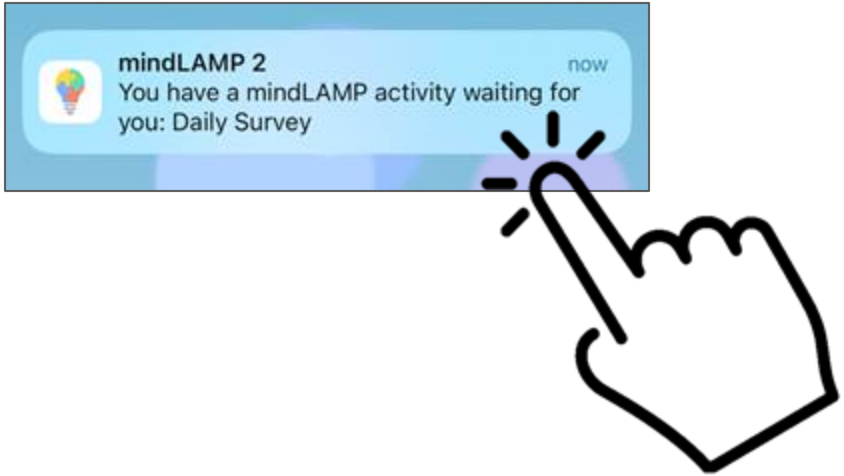

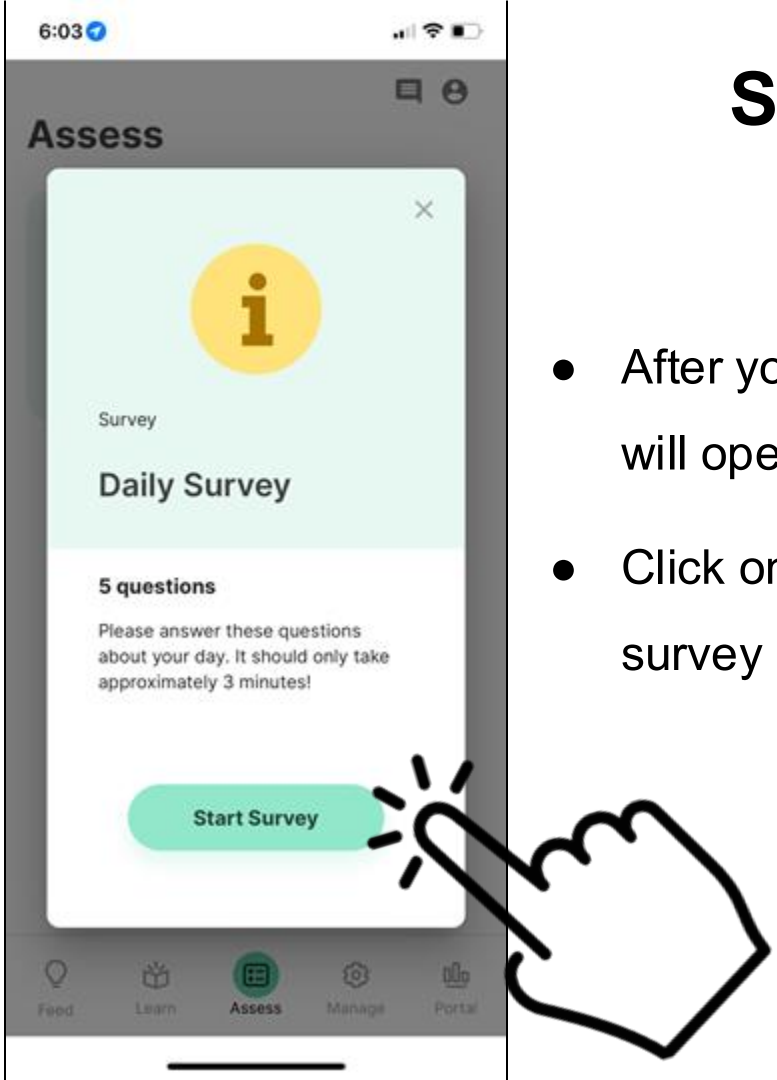

Step 1: Start Survey

- After you tap the notification, the mindLAMP app will open and will take you to the Daily Survey
- Click on the “Start Survey” button to begin the survey

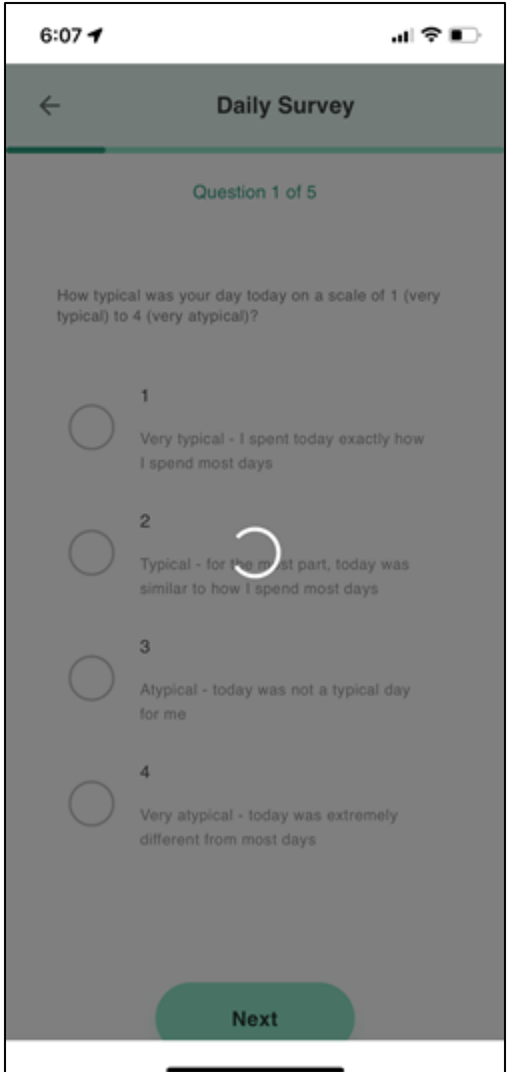

Step 1: Start Survey

- The screen may freeze for a few seconds as it loads the survey -- that is OK, just wait for the survey to appear

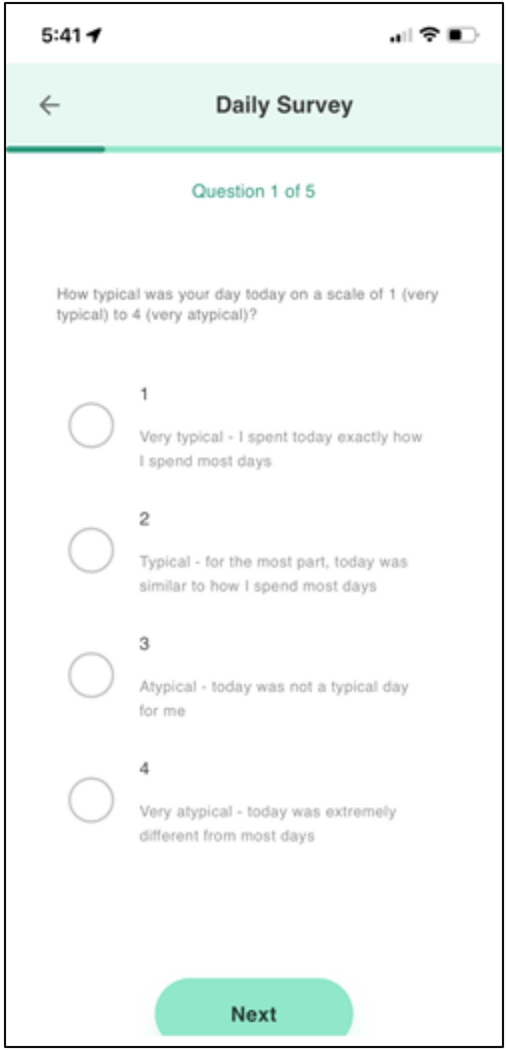

Step 2: Answer survey questions

- The first question will ask you **how typical your day was**, on a scale of *1 (very typical)*, to *4 (very atypical)*.

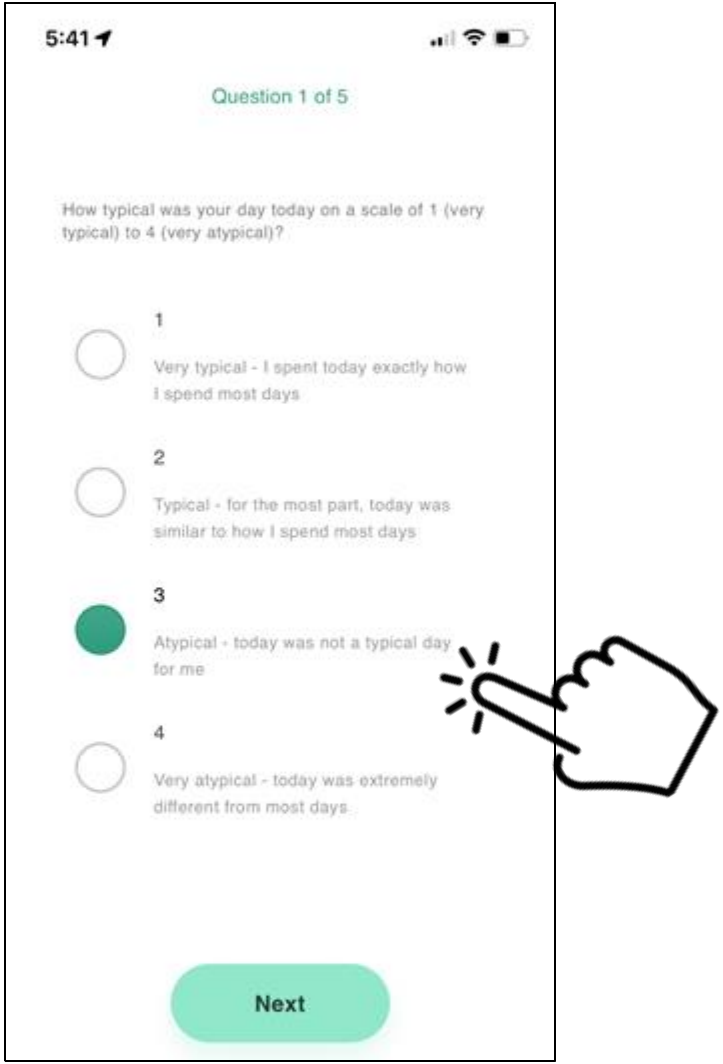

Step 2: Answer survey questions

- Select the circle that corresponds to your answer; it will turn green after you've made your choice
- Click "Next" at the bottom to proceed
- *Hint: you may need to scroll down to access the "Next" button*

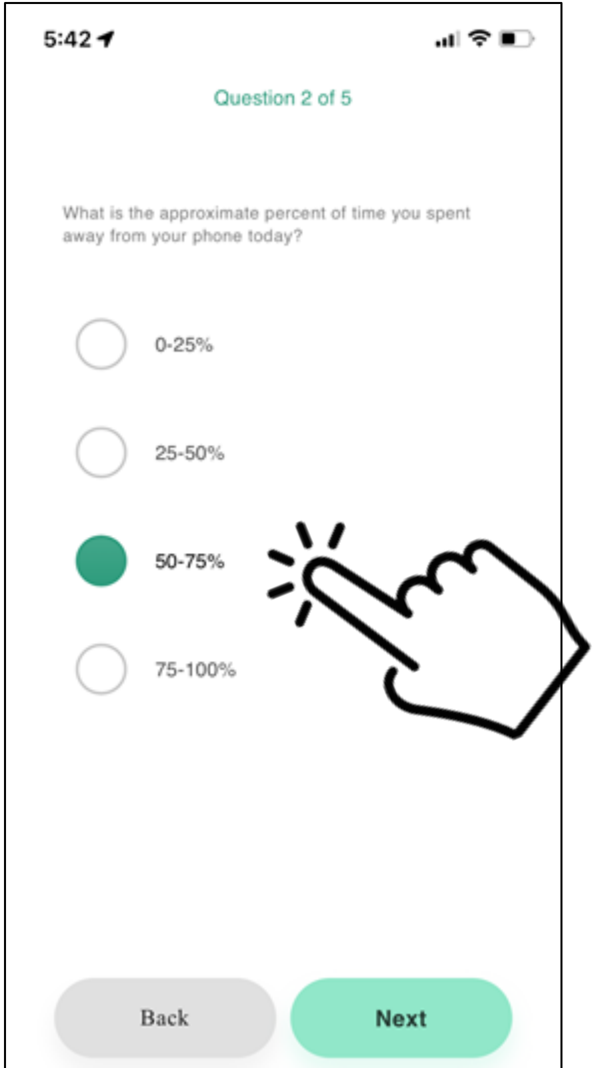

Step 2: Answer survey questions

- The second question will ask you to **<u>estimate</u> what percent of the day you spent away from your phone**
- Again, select the circle that corresponds to your answer; it will turn green after you've made your choice
- Click "Next" at the bottom to proceed, or click "Back" to revise your response to the previous question

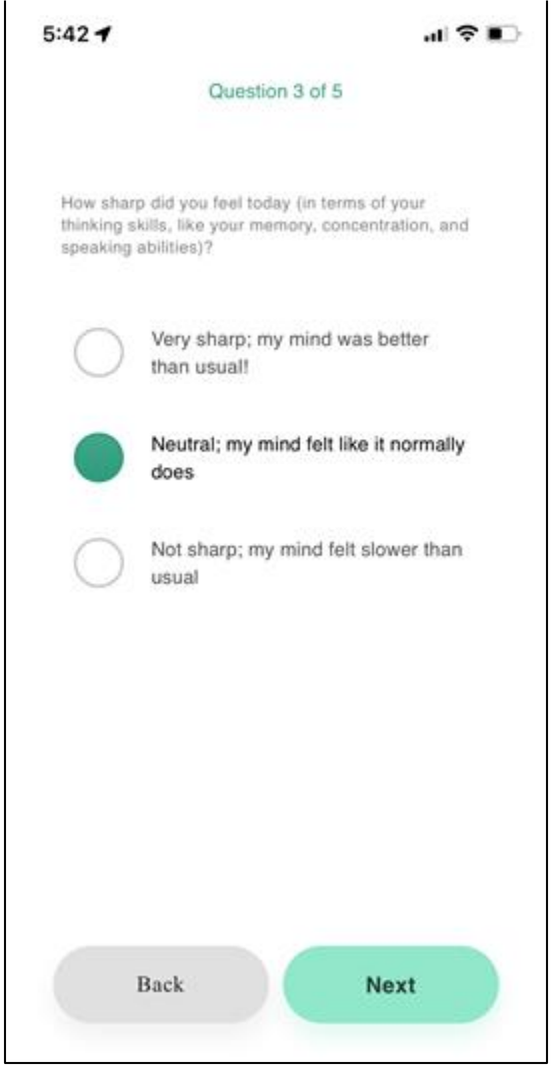

Step 2: Answer survey questions

- The third question will ask **how sharp you felt today** (in terms of your thinking skills, like your memory, concentration, and speaking abilities), on a scale from *very sharp* to *not sharp*
- Again, select the circle that corresponds to your answer; it will turn green after you've made your choice
- Click "Next" at the bottom to proceed, or click "Back" to revise your response to the previous question

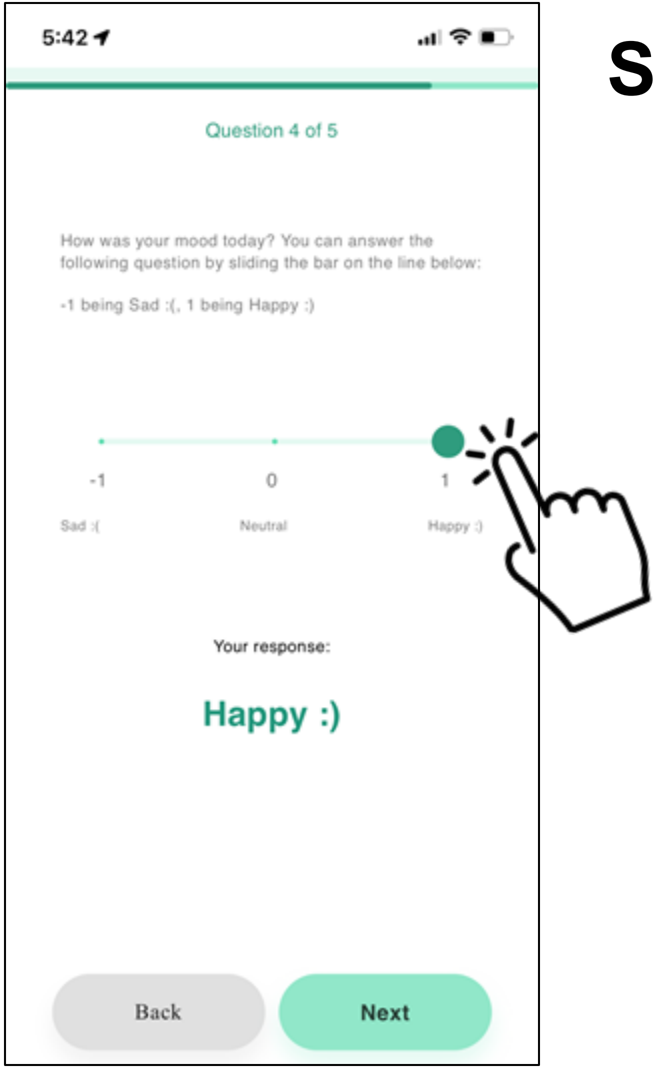

Step 2: Answer survey questions

- The fourth question will ask **how your mood was today**, on a scale from *-1 (Sad)*, *0 (neutral)*, to *1 (Happy)*
- This time, tap the scale and drag the green circle to your response choice. Your selected response will appear in writing below.
- Click “Next” at the bottom to proceed, or click “Back” to revise your response to the previous question

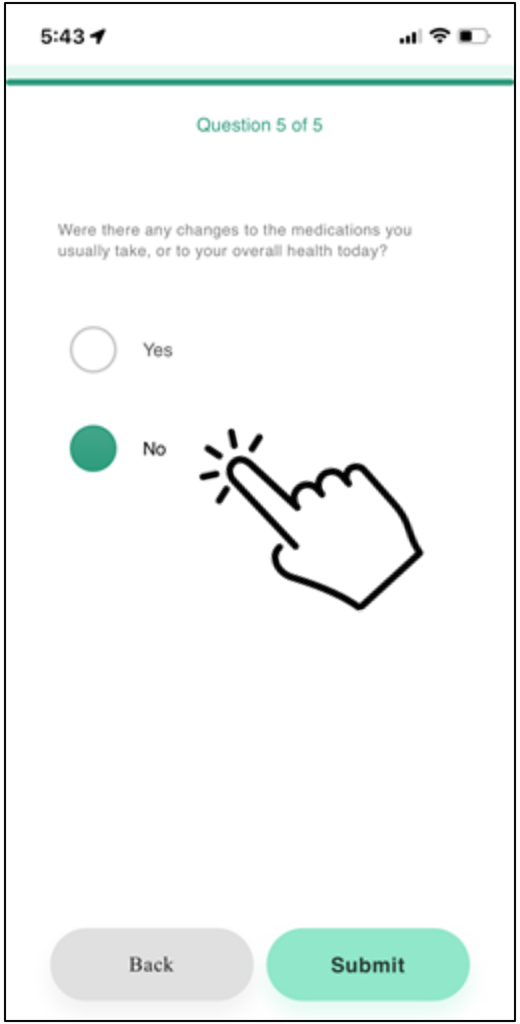

Step 2: Answer survey questions

- The final question will ask if there were any **changes to your medication or health today**. Simply answer *"Yes"* or *"No"*
- Click "Submit" at the bottom to complete your survey!

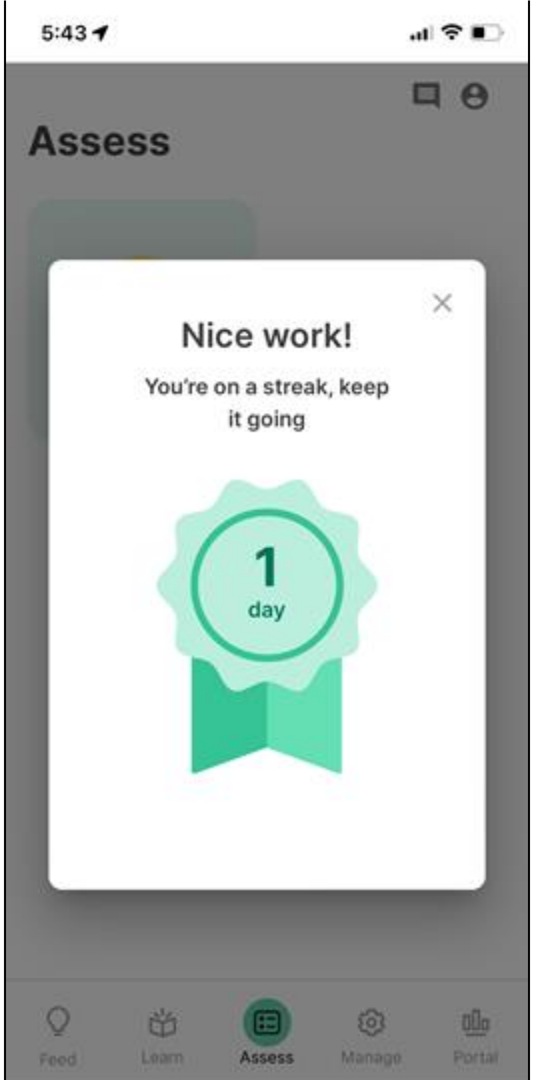

Step 2: Answer survey questions

- Once you have successfully completed the survey, you should see a note congratulating you on your completion.
- It will keep track of how many days in a row you have completed the survey - the higher the better!
- Close out of mindLAMP by pressing your home button or swiping up on the screen; do NOT log out

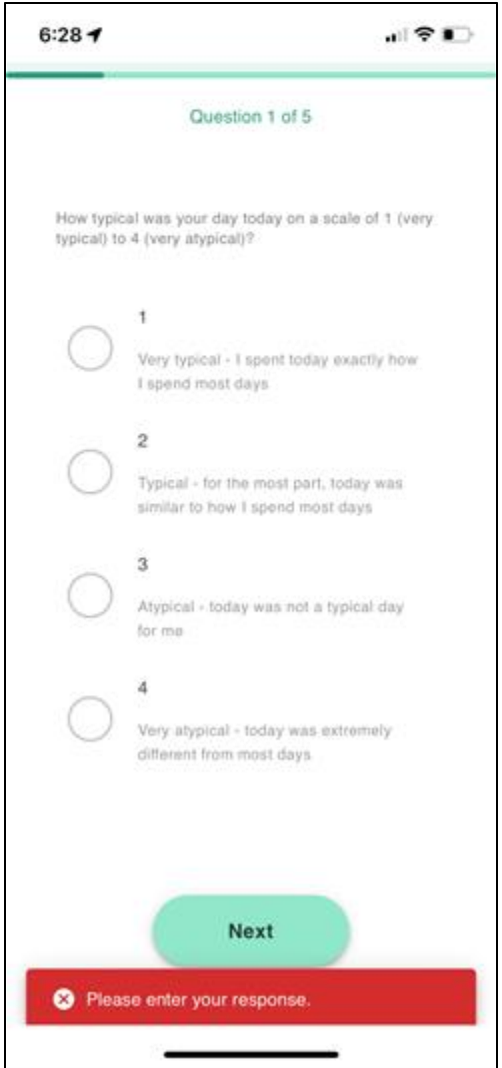

Step 2: Answer survey questions

- **Warning!** If you try to submit a question without providing a response, you will see a message in red at the bottom of the screen.
- Select a response before hitting "next"

What else is available in mindLAMP?

- When you click on the mindLAMP app from your homescreen, you will be taken to the **Assess** tab
- You can complete the Daily Survey from here if you miss the survey notification
- Do NOT complete the Daily Survey before the nightly notification time; wait until the end of each day to complete the Daily Survey

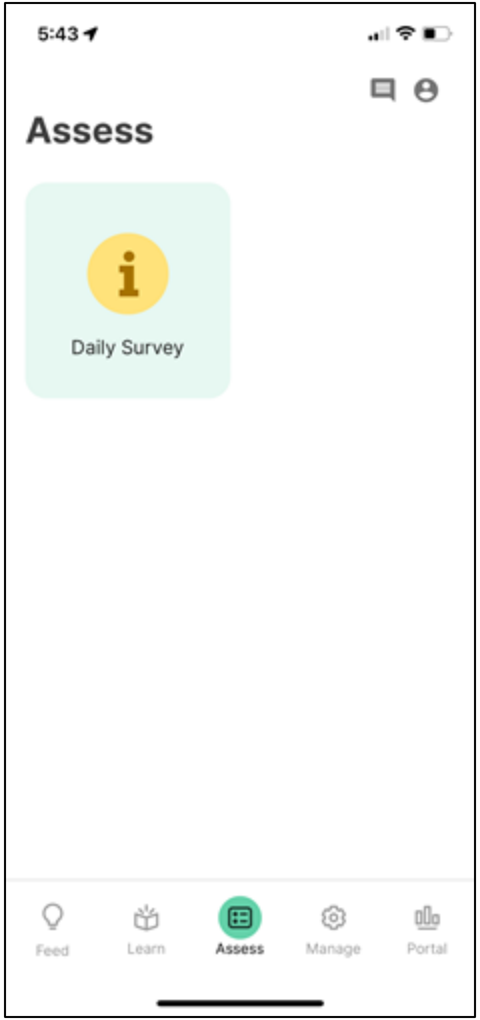

What else is available in mindLAMP?

- The “**Feed**” tab will simply display that you have a scheduled survey each night

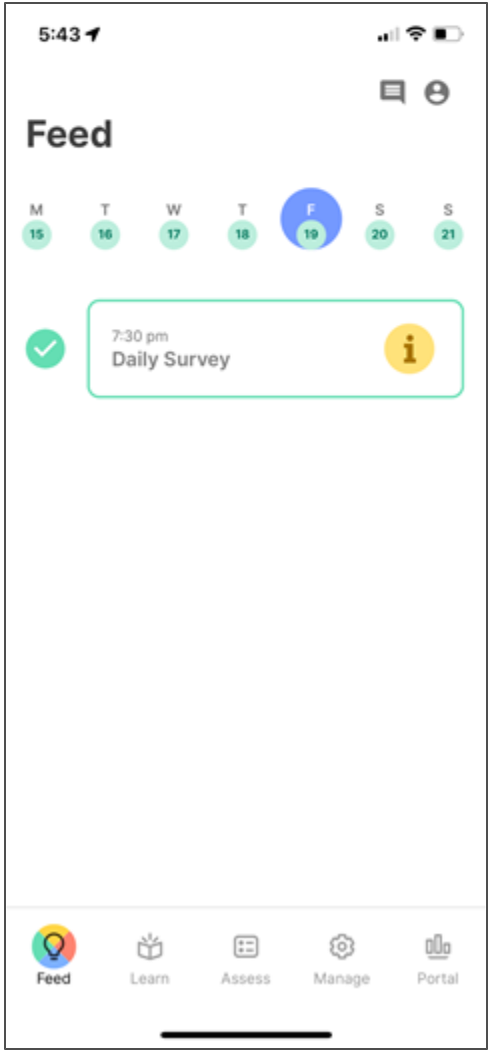

Now you try it…

1) **Pretend it is 10 pm and you missed the survey notification**
2) **Tap on the mindLAMP app on your phone**
3) **Select Daily Survey**
4) **Complete each survey question**
5) **Submit the survey**
6) **Exit the app without logging out**

Tell us the steps you are doing out loud as you do them

**Experimenter: note the number of times you repeated this step*

Just a few more points...

- What to do if you miss a survey
- What to do if you get logged out
- Other notifications you may see
- Important troubleshooting reminders
- What to do if you have trouble

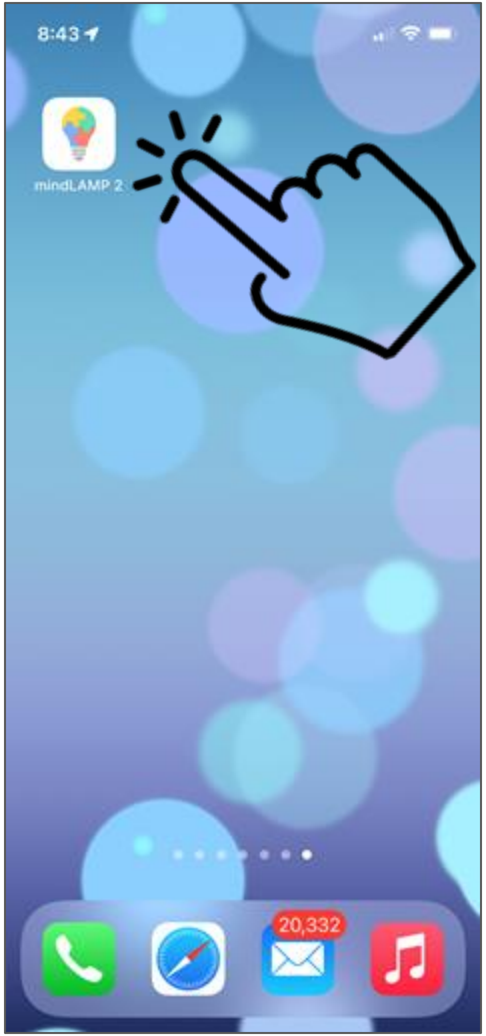

What to do if you miss a survey

- Click on the mindLAMP app on your home screen
- Click on “Daily Survey” within the Assess Tab, and complete the survey for that day
- Only do this if you missed the nightly survey notification; do NOT complete the survey until the end of your day
- You will not be able to complete surveys for previous, or future days

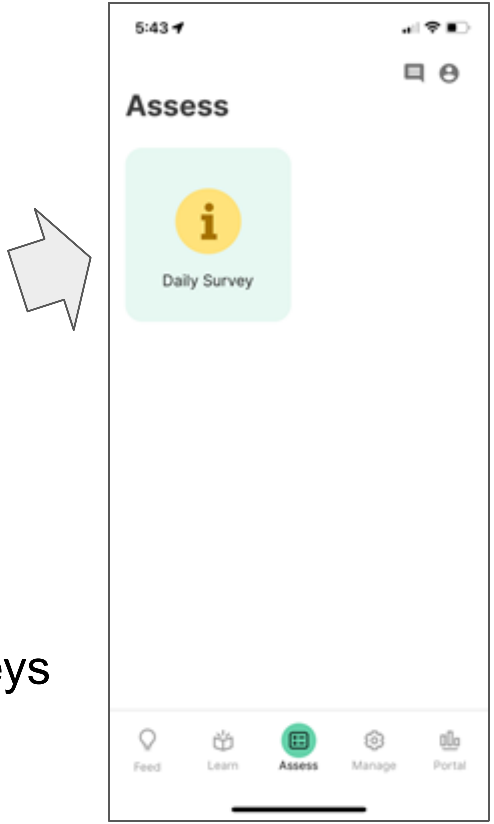

What to do if you are logged out

If, after clicking on the survey notification or the on the mindLAMP app, you are sent to the login screen …

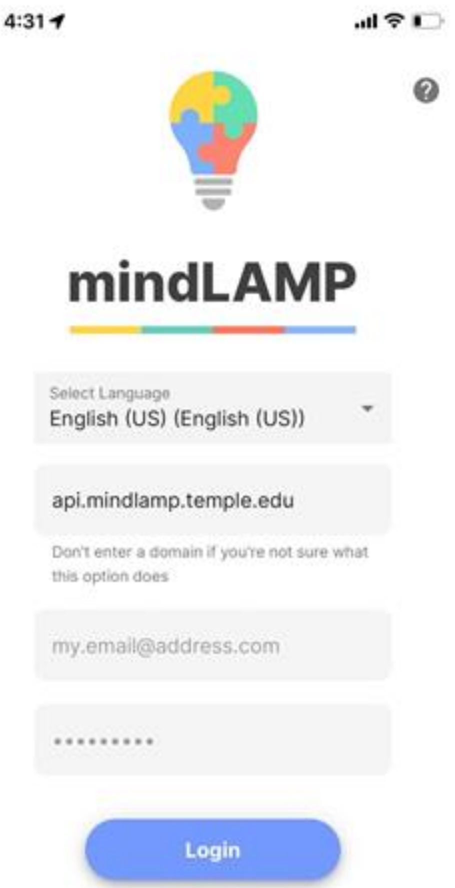

This is a technical glitch. To work around this, first try…
[examiner demonstrate, then practice together]

1) Closing out of the app

  a) Phones with a home button: unlock phone > double tap home button > locate the mindLAMP app > flick up

  a) Phones without a home button: unlock phone > slowly swipe up and let go > locate the mindLAMP app > flick up

1) Re-launching the app

  a) Find the mindLAMP app icon on your home screen & click on it

  b) Click on "Daily Survey"

What to do if you are logged out

If, after closing and relaunching the app, you are still logged out…

Enter the information below into the fields seen on the right.

api.mindlamp.temple.edu

U……@lamp.com**

TEMPLE

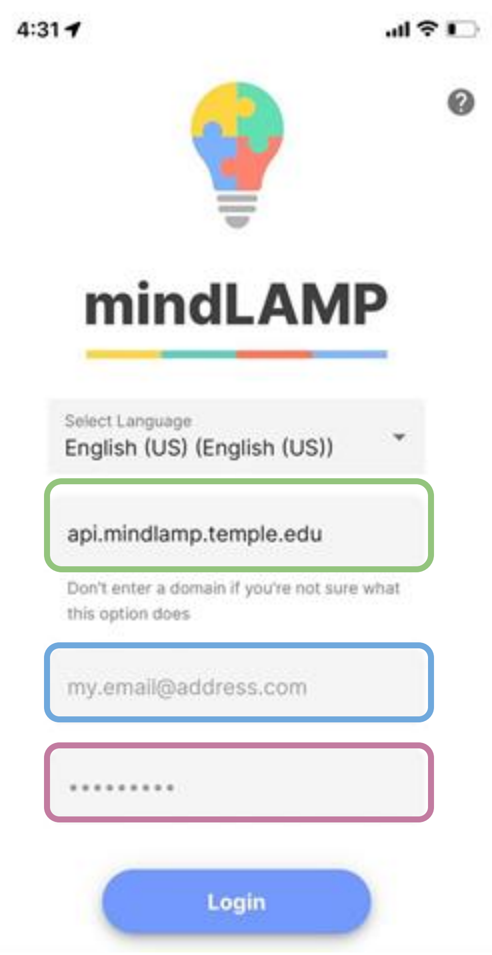

****use the login handout in your study binder for your unique login info**

Other notifications you may see

- You may see a notification like the one on the right
- This is a reminder that mindLAMP is collecting GPS data
- When you see this notification, make sure you select "**Always Allow**"
- If you do not select "**Always Allow**", the app will not collect sufficient data for the study
- Once you complete the study, the app will be deleted, GPS data will not be collected and you will no longer see notifications like this

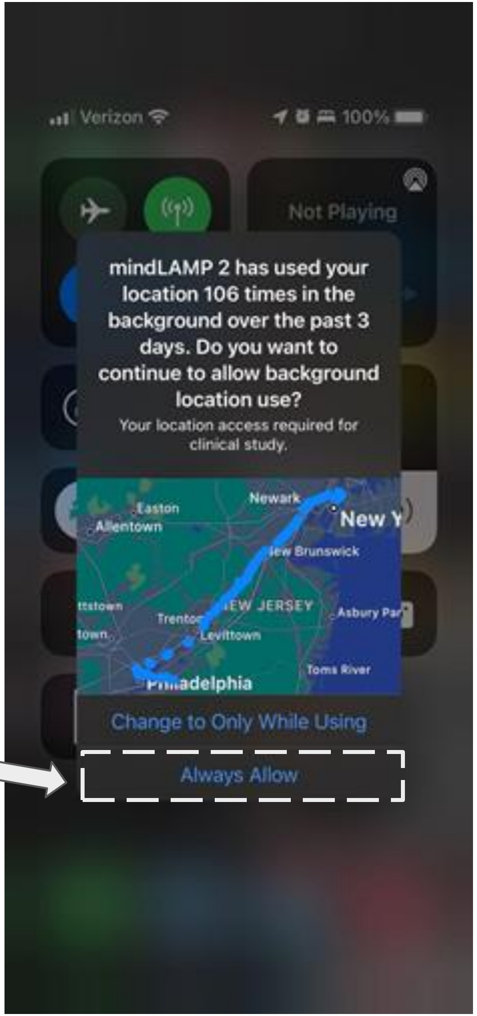

Troubleshooting Reminders

- Make sure your phone is always charged
- If your phone is in low battery mode, charge your phone

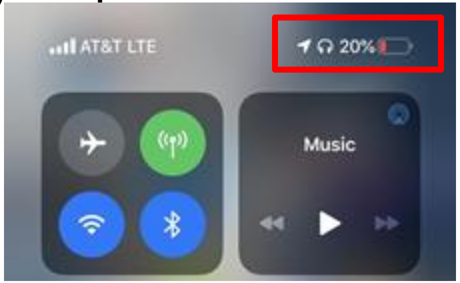

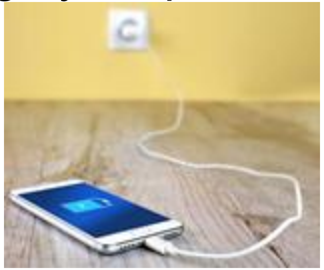

- NEVER to go into low power mode or airplane mode

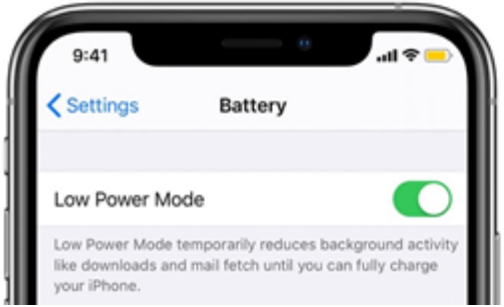

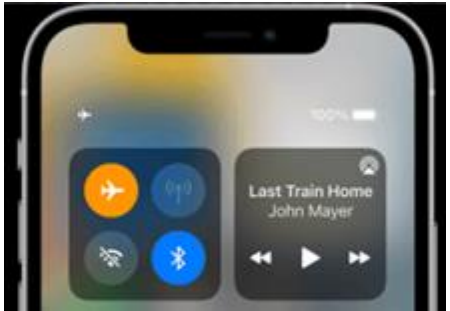

Troubleshooting Reminders

- When using the mindLAMP app, do NOT ever log out
- If you want to leave the app, simply press your home button or swipe up on your screen to exit the app

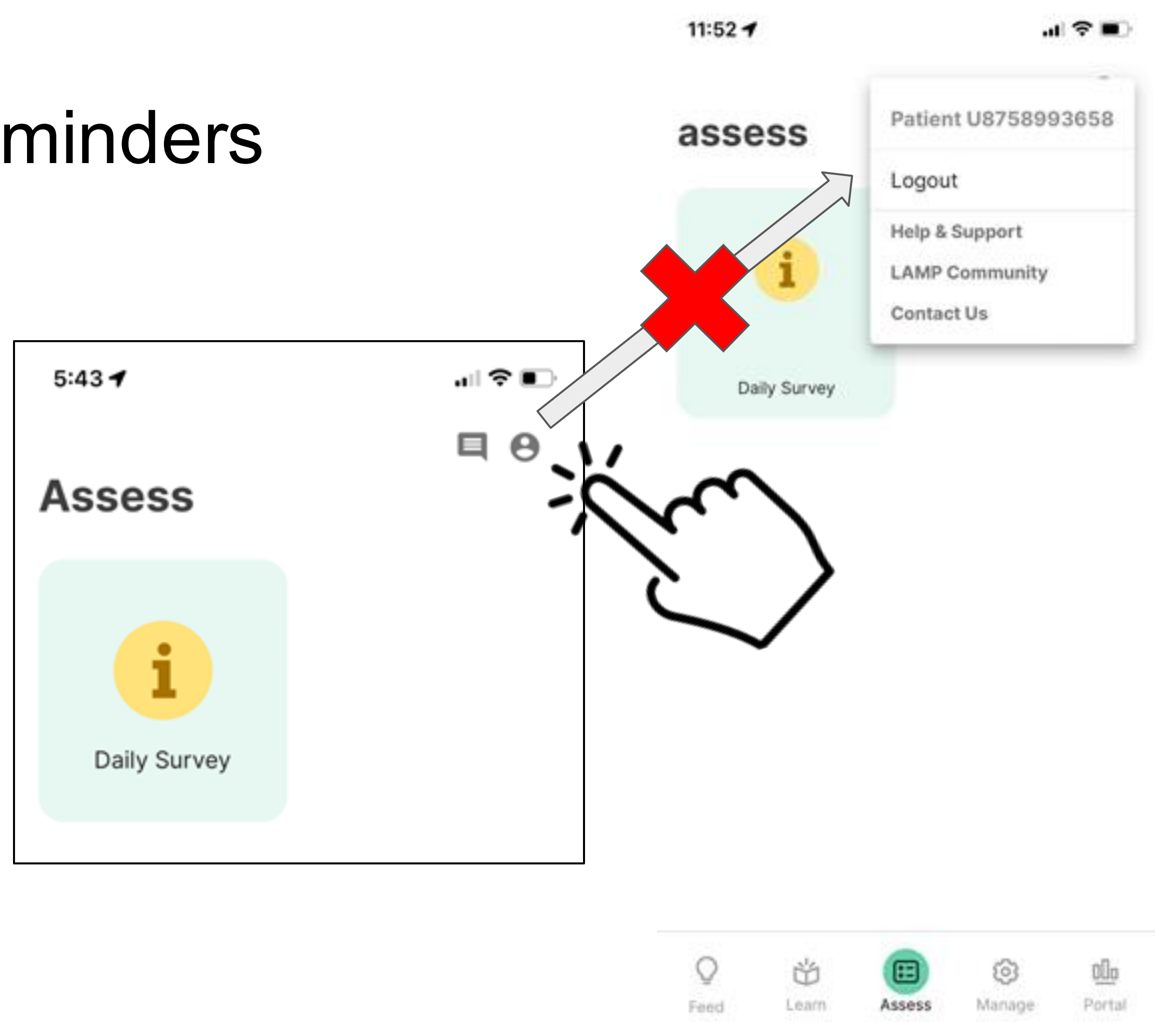

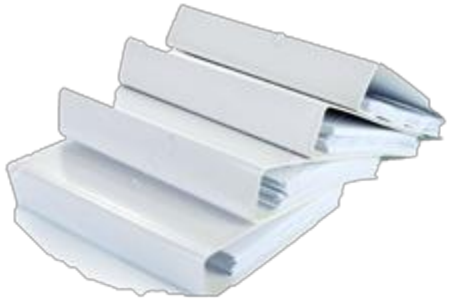

Troubleshooting Steps

- ❑ Is low power mode turned off?
- ❑ Is airplane mode turned off?
- ❑ Is the device consistently connected to WiFi?
- ❑ Is the device powered on at all times of the day?
- ❑ Are all permissions granted for mindLAMP in the Settings app?
- ❑ Are you logged in to mindLAMP?
- ❑ Have you tried closing out and relaunching the app?

Look in the study binder for directions.

Still Need Help? Contact us any time.

Email: aging@temple.edu

Phone number: **(484)-843-1321**

Examiner: record end time
(once training is completed)

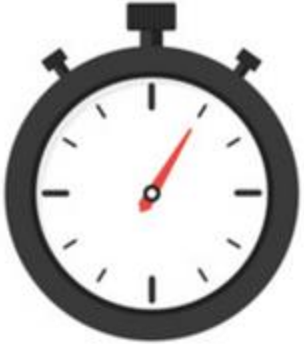

Using the mindLAMP App -- *Androids*

Participant & Informant Training Procedures

Examiner: record start time
(& whether in-person or via zoom)

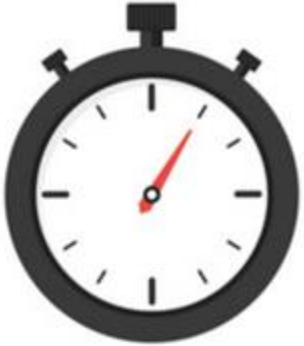

Basic Functions

The mindLAMP application is designed to:

1. Collect de-identified data from your phone sensors throughout the day without any interference while you go about your regular daily activities.

2. Collect responses to a brief 5-question survey. The survey should take about **3 minutes** to complete.

This is what the app will look like on your phone screen.
(show on participant's phone)

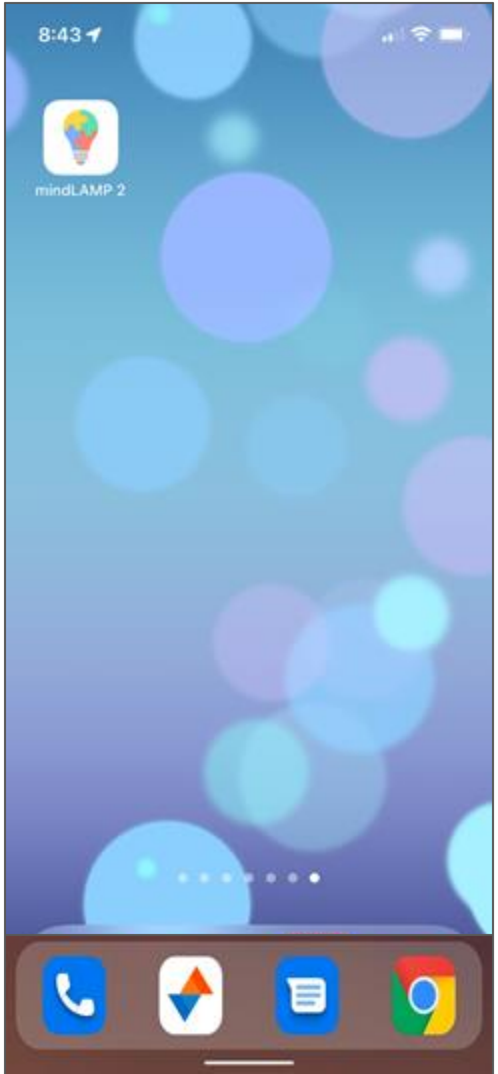

Nightly survey schedule

You will receive an alarm notification to complete the survey at the same time, each night, for the next 4 weeks.

Your nightly notification is scheduled for ________ pm; it will be displayed from your phone's alarm clock app

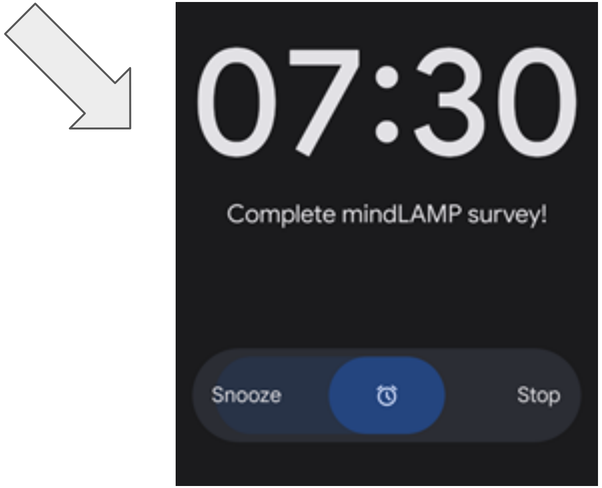

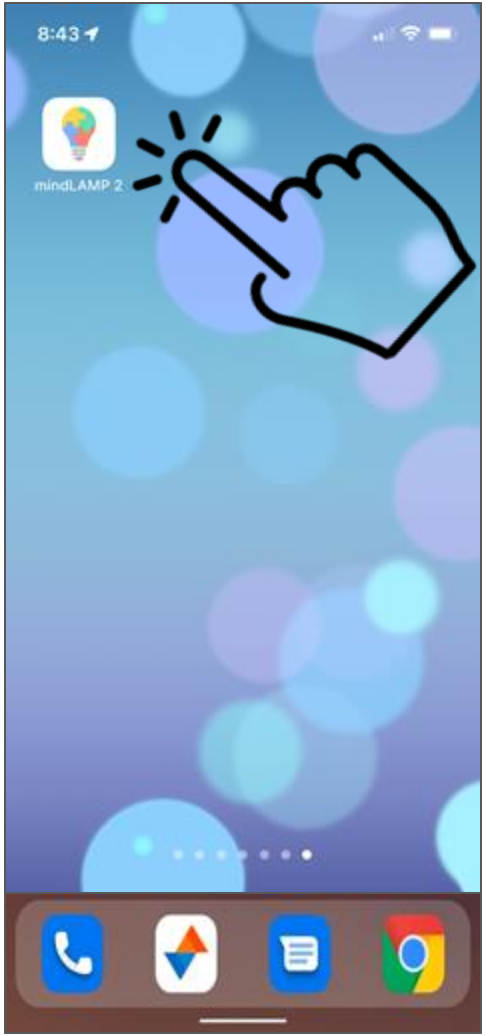

Responding to the nightly survey

- Once your nightly alarm goes off, find the mindLAMP app on your home screen and click on it (If your phone is locked, you'll need to unlock your phone first, and then click on the mindLAMP app)
- Next, click on "Daily Survey" within the **Assess** Tab, and complete the survey for that day
- Only do this once your nightly alarm goes off; do NOT complete the survey until the end of your day
- You will not be able to complete surveys for previous, or future days

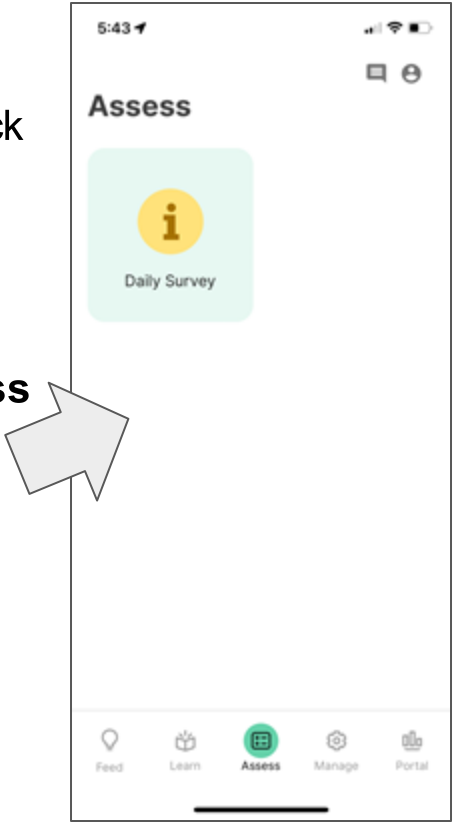

Responding to the nightly survey

If you see a pop-up asking you to “Choose an account” after clicking on the mindLAMP app,

Click on the account associated with your Google Fit login

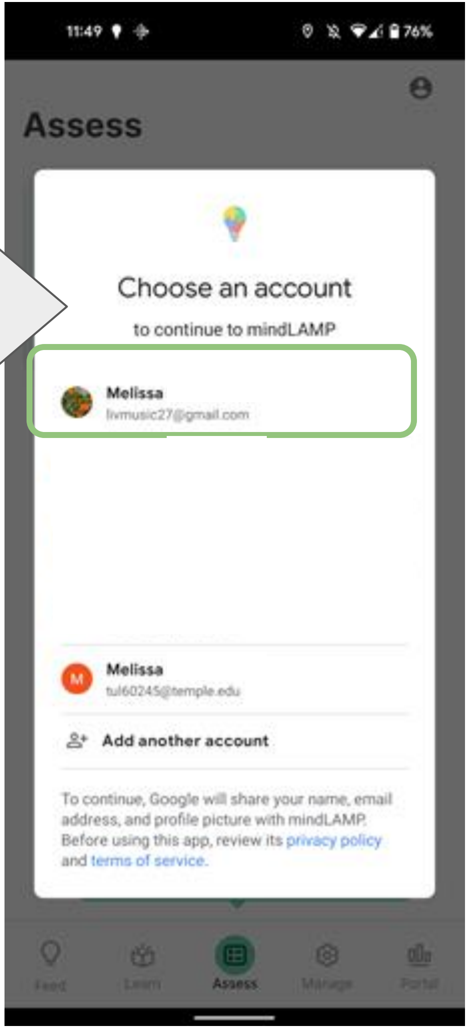

You will then be taken to the mindLAMP Assess tab, where you will find the Daily Survey button

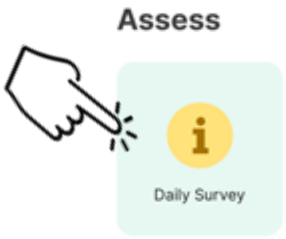

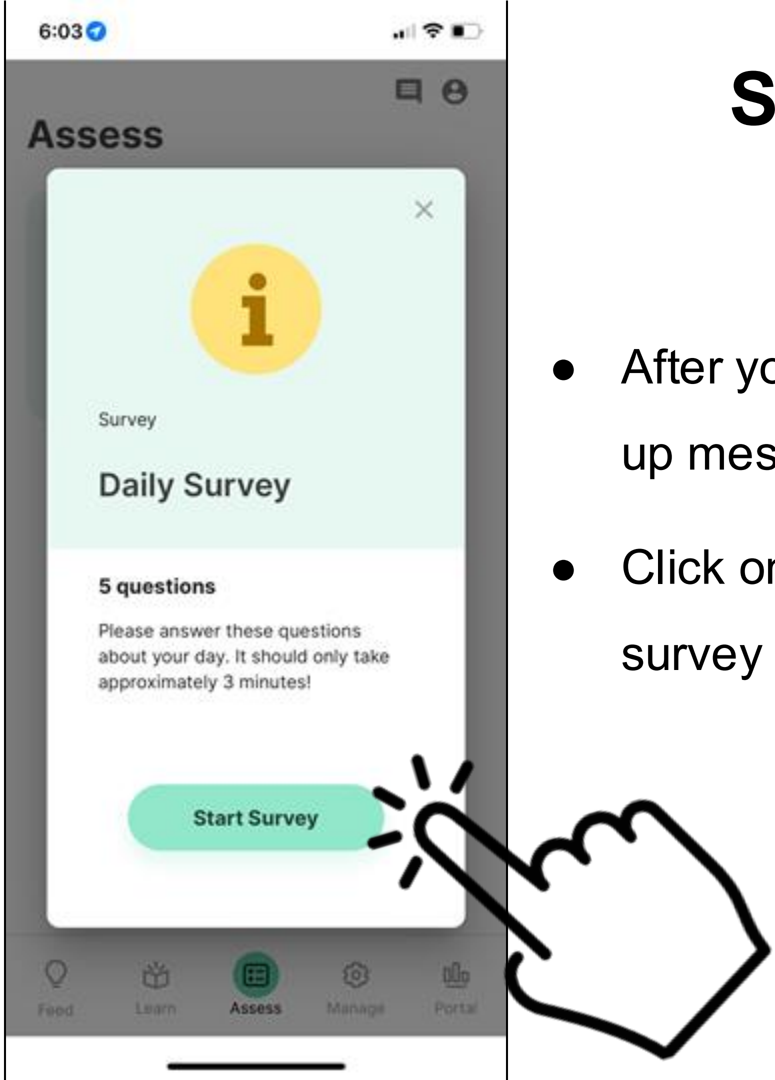

Step 1: Start Survey

- After you tap on “Daily Survey”, you will see a pop-up message
- Click on the “Start Survey” button to begin the survey

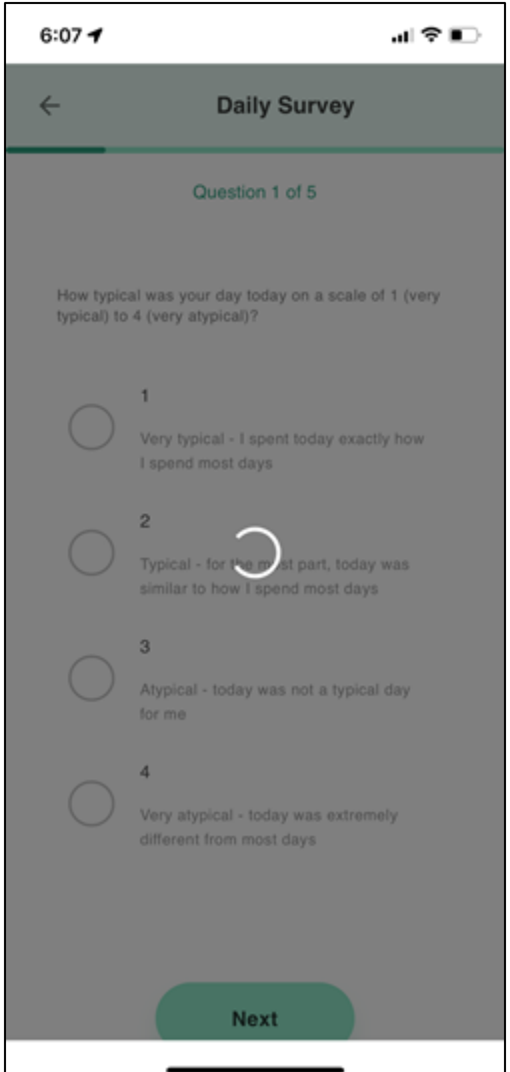

Step 1: Start Survey

- The screen may freeze for a few seconds as it loads the survey -- that is OK, just wait for the survey to appear

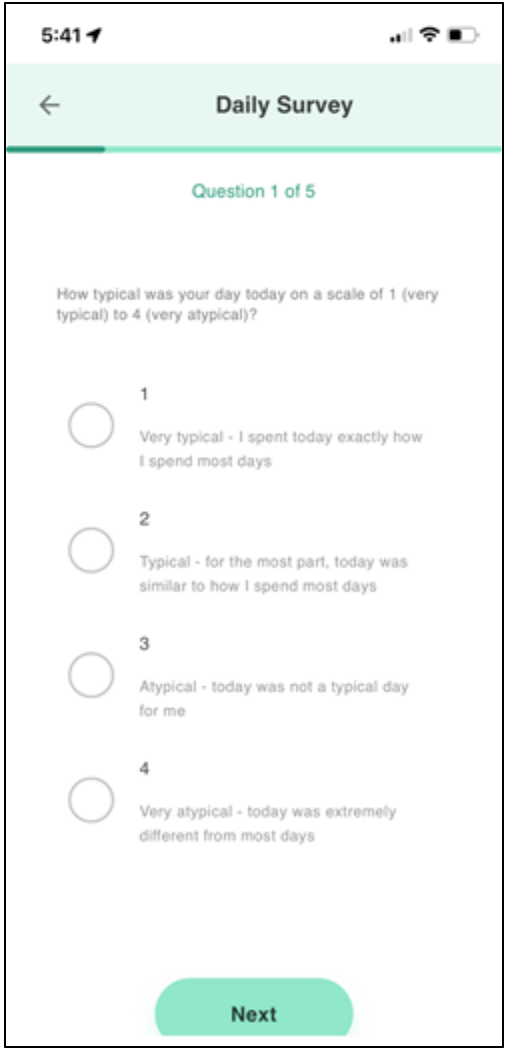

Step 2: Answer survey questions

- The first question will ask you **how typical your day was**, on a scale of *1 (very typical)*, to *4 (very atypical)*.

Step 2: Answer survey questions

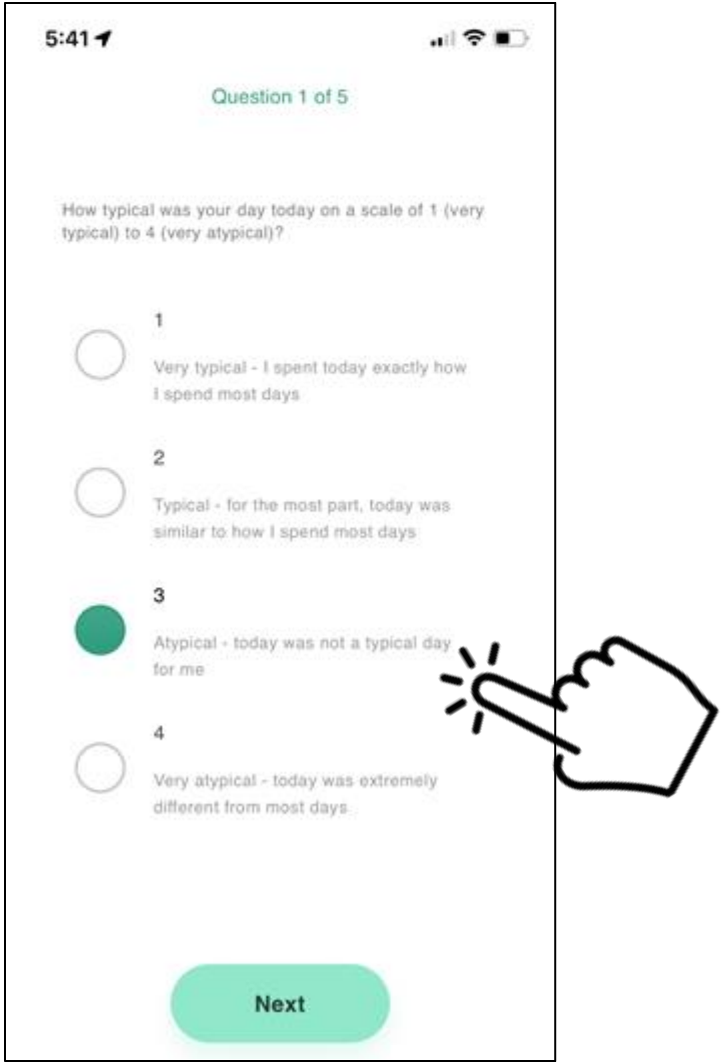

- Select the circle that corresponds to your answer; it will turn green after you've made your choice
- Click "Next" at the bottom to proceed
- *Hint: you may need to scroll down to access the "Next" button*

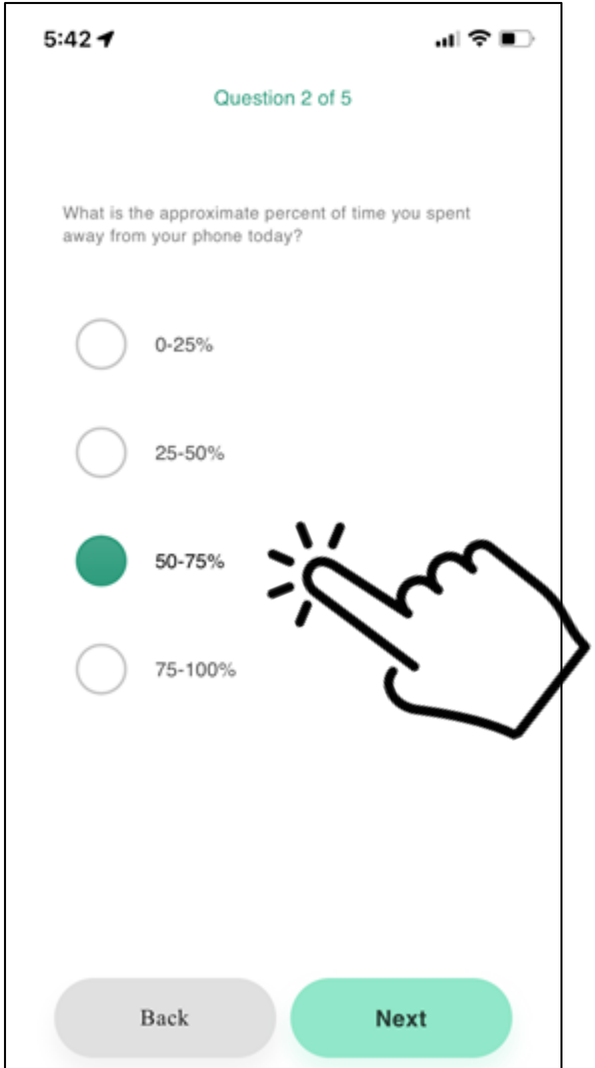

Step 2: Answer survey questions

- The second question will ask you to **<u>estimate</u> what percent of the day you spent away from your phone**
- Again, select the circle that corresponds to your answer; it will turn green after you've made your choice
- Click "Next" at the bottom to proceed, or click "Back" to revise your response to the previous question

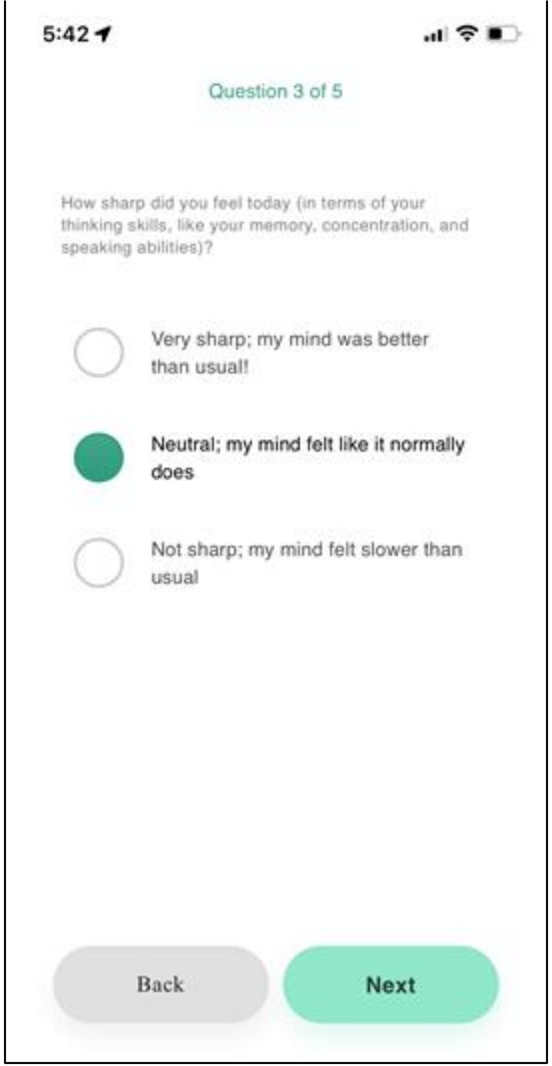

Step 2: Answer survey questions

- The third question will ask **how sharp you felt today** (in terms of your thinking skills, like your memory, concentration, and speaking abilities), on a scale from *very sharp* to *not sharp*
- Again, select the circle that corresponds to your answer; it will turn green after you've made your choice
- Click "Next" at the bottom to proceed, or click "Back" to revise your response to the previous question

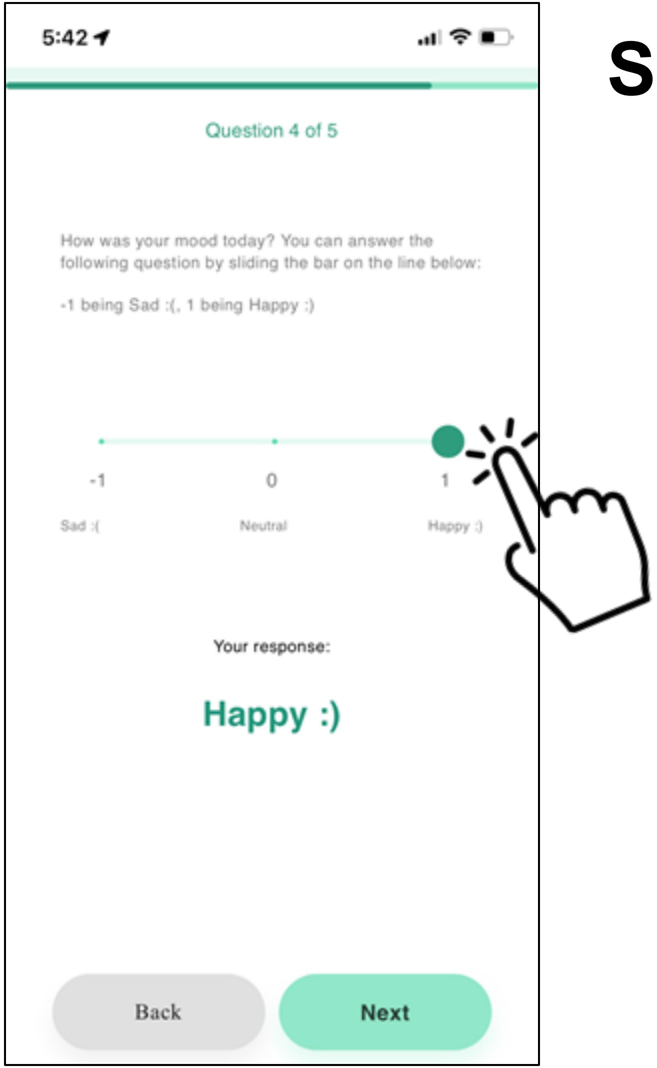

Step 2: Answer survey questions

- The fourth question will ask **how your mood was today**, on a scale from *-1 (Sad)*, *0 (neutral)*, to *1 (Happy)*
- This time, tap the scale and drag the green circle to your response choice. Your selected response will appear in writing below.
- Click “Next” at the bottom to proceed, or click “Back” to revise your response to the previous question

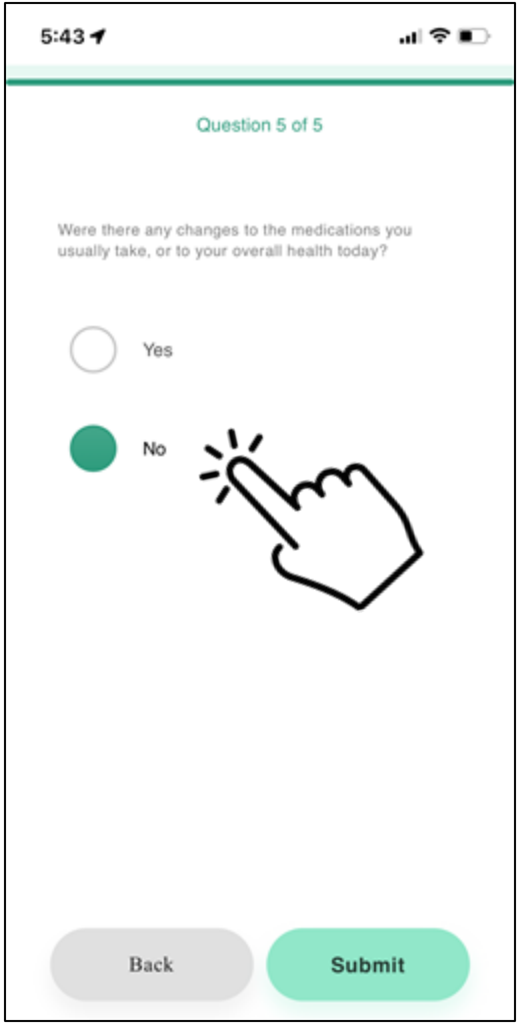

Step 2: Answer survey questions

- The final question will ask if there were any **changes to your medication or health today**. Simply answer *“Yes”* or *“No”*
- Click “Submit” at the bottom to complete your survey!

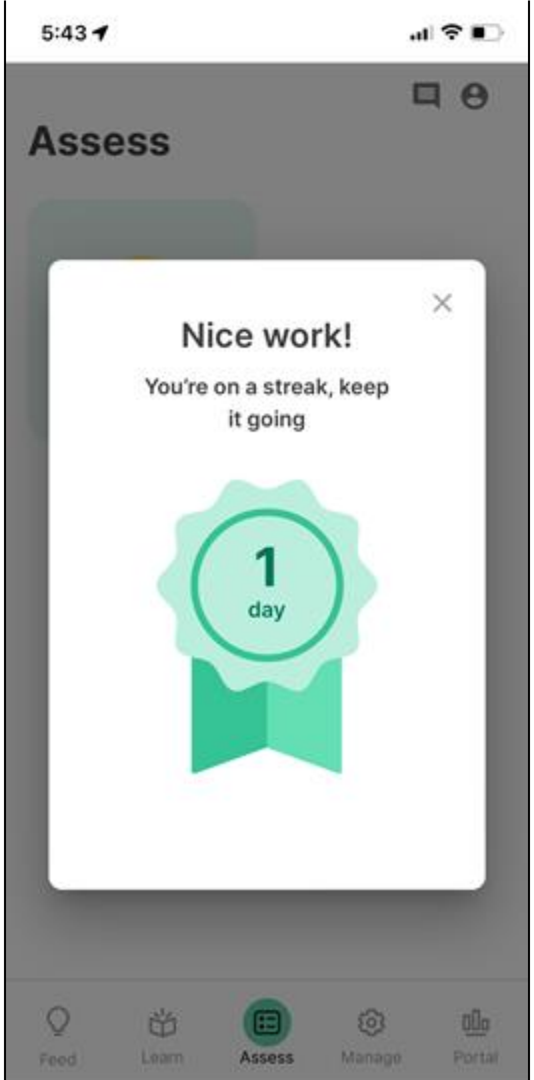

Step 2: Answer survey questions

- Once you have successfully completed the survey, you should see a note congratulating you on your completion.
- It will keep track of how many days in a row you have completed the survey - the higher the better!
- Close out of mindLAMP by pressing your home button or swiping up on the screen; do NOT log out

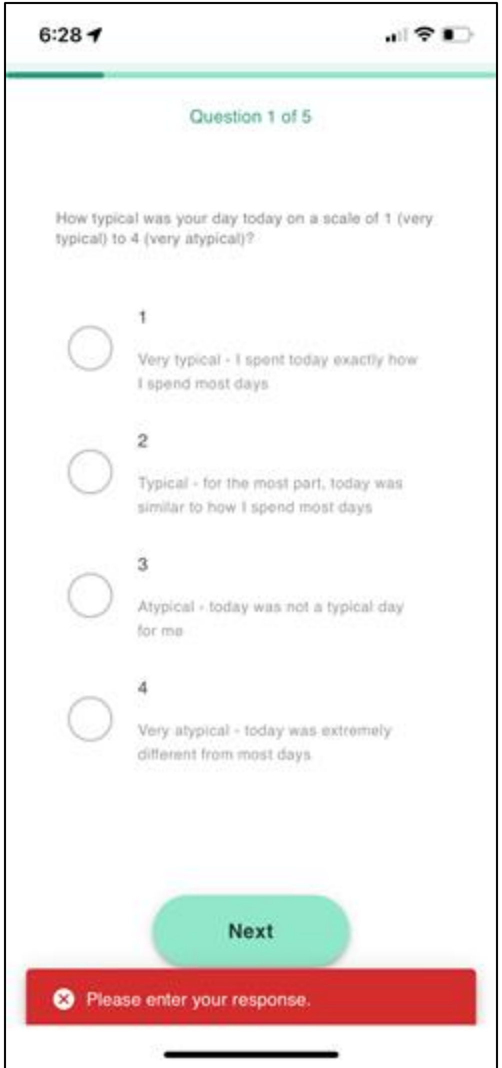

Step 2: Answer survey questions

- **Warning!** If you try to submit a question without providing a response, you will see a message in red at the bottom of the screen.
- Select a response before hitting “next”

What else is available in mindLAMP?

- When you click on the mindLAMP app from your homescreen, you will be taken to the **Assess** tab
- You can complete the Daily Survey from here if you miss the survey alarm
- Do NOT complete the Daily Survey before the nightly alarm; wait until the end of each day to complete the Daily Survey

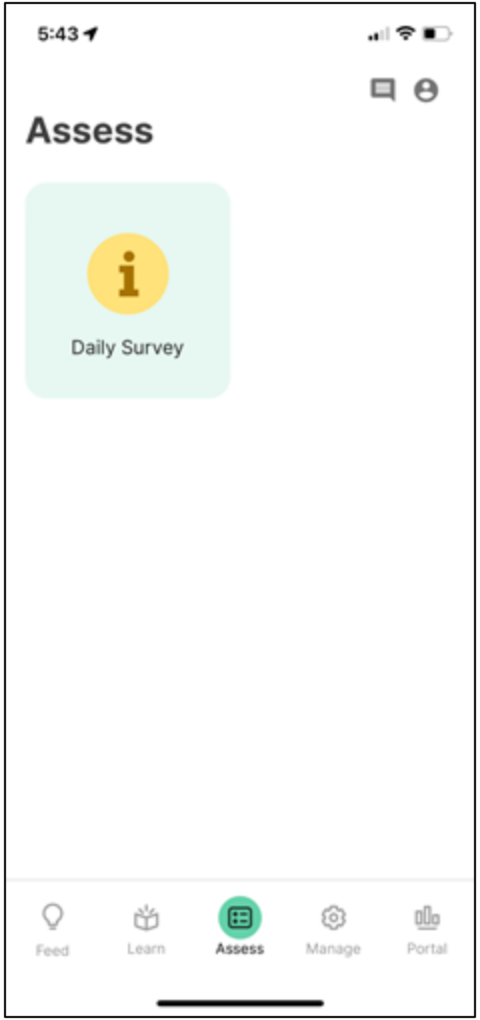

What else is available in mindLAMP?

- The "**Feed**" tab will simply display that you have a scheduled survey each night

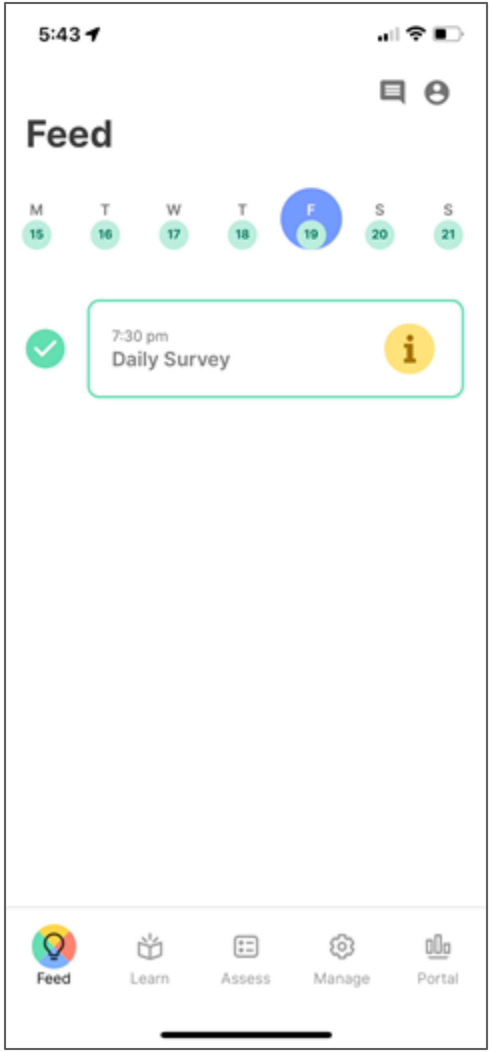

Now you try it…

1) **Pretend you just received your nightly survey alarm**
2) **Tap on the mindLAMP app on your phone**
3) **Select your Google Fit account (if you see the pop-up)**
4) **Select Daily Survey**
5) **Complete each survey question**
6) **Submit the survey**
7) **Exit the app without logging out**

Tell us the steps you are doing out loud as you do them

**Experimenter: note the number of times you repeated this step*

Just a few more points...

- What to do if you miss a survey
- What to do if you get logged out
- Other notifications you may see
- Important troubleshooting reminders
- What to do if you have trouble

What to do if you miss a survey

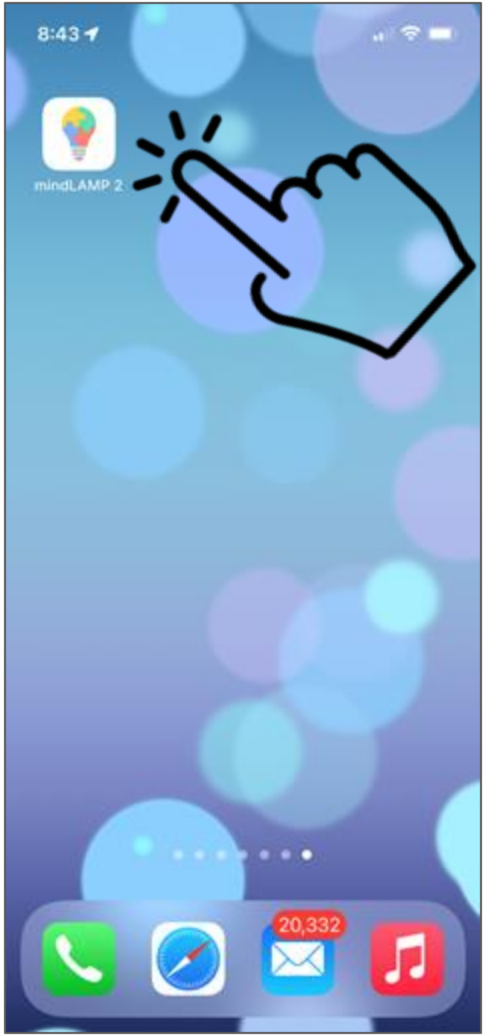

- Click on the mindLAMP app on your home screen
- Click on “Daily Survey” within the Assess Tab, and complete the survey for that day
- Only do this if you missed the nightly survey notification; do NOT complete the survey until the end of your day
- You will not be able to complete surveys for previous, or future days

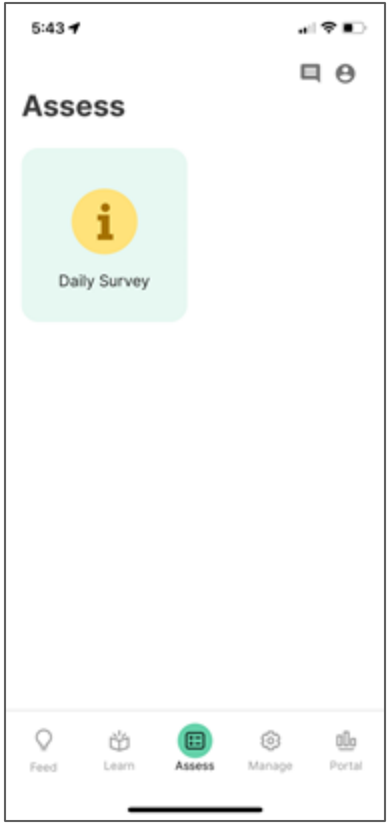

What to do if you are logged out

If, after clicking on the mindLAMP app, you are sent to the login screen …

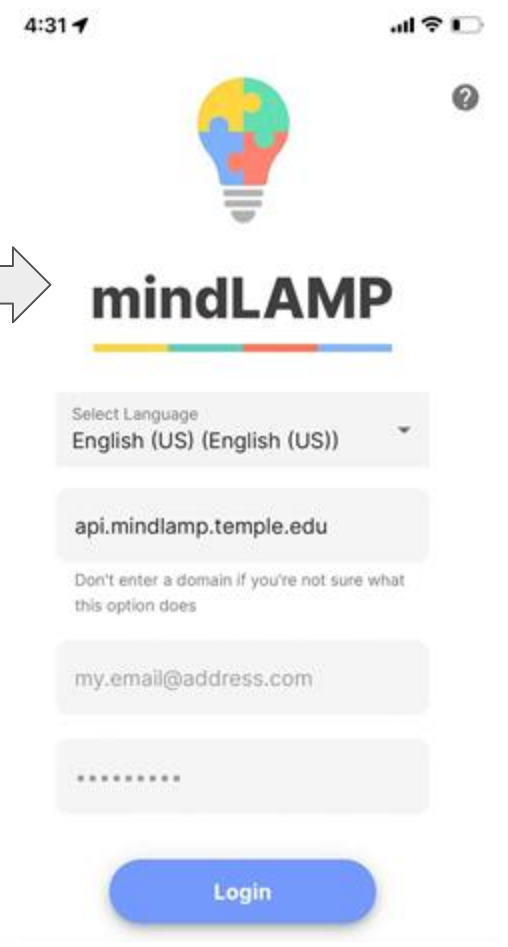

This is a technical glitch. To work around this:
[examiner demonstrate, then practice together]

1) First close out of the app
   a) Swipe up and hold from the bottom of your phone screen to open the recent apps menu.
   b) Within the recent apps, locate mindLAMP.
   c) Depending on the phone, you'll have to swipe up, down, left, or right to close the app.

1) Then re-launch the app
   a) Find the mindLAMP app icon on your home screen & click on it
   b) Click on "Daily Survey" to complete the survey

What to do if you are logged out

If, after closing and relaunching the app, you are still logged out…

Enter the information below into the fields seen on the right.

api.mindlamp.temple.edu

U……@lamp.com**

TEMPLE

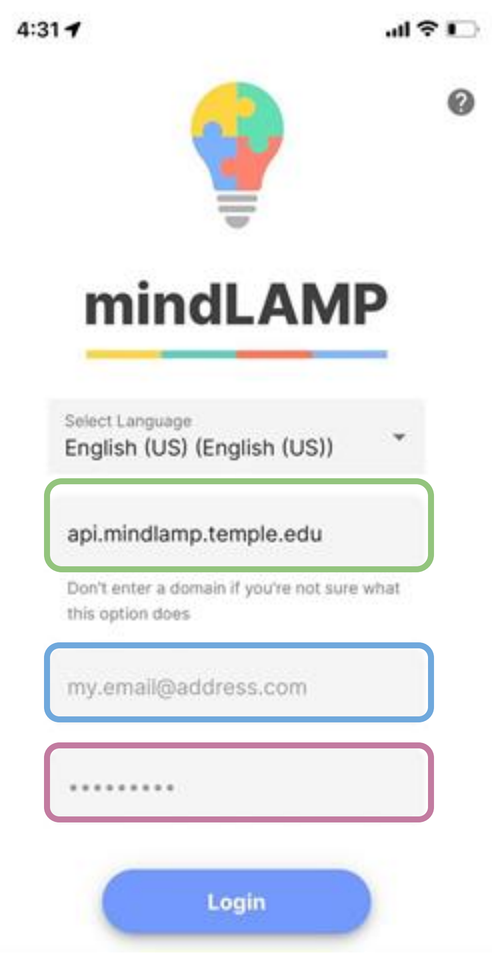

****use the login handout in your study binder for your unique login info**

Other notifications you may see

- You may see a notification like the one on the right
- This is a reminder that mindLAMP is collecting GPS data
- When you see this notification, make sure you select "**Always Allow**"
- If you do not select "**Always Allow**", the app will not collect sufficient data for the study
- Once you complete the study, the app will be deleted, GPS data will not be collected and you will no longer see notifications like this

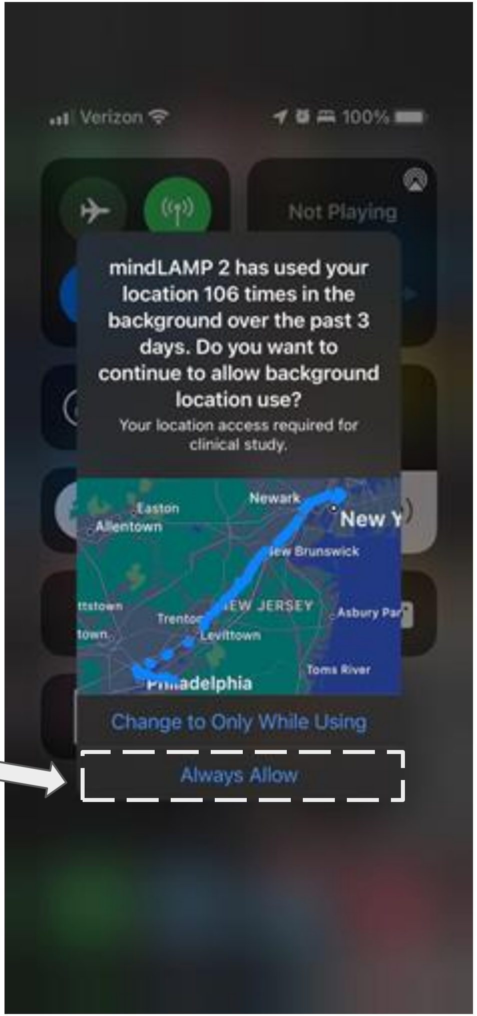

Other notifications you may see

- You may occasionally see a notification like the one on the right when you open the mindLAMP app. If this occurs, please close out and re-launch the app (using the steps outlined in this slideshow)
- If the notification continues to display, please try to re-launch the app the following day

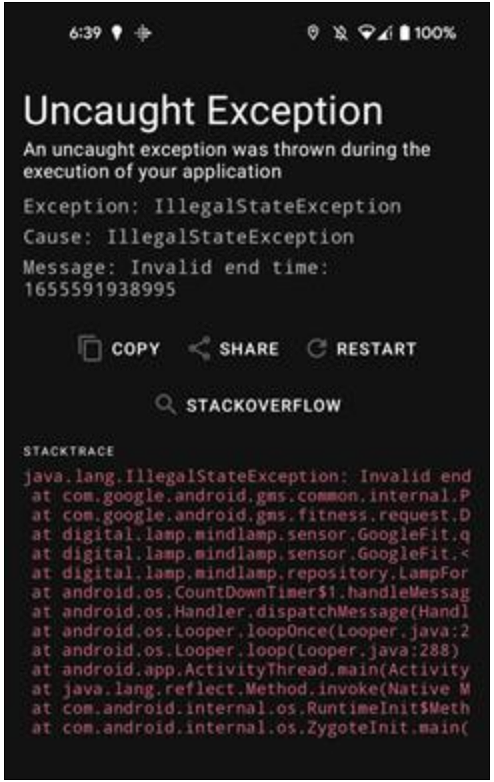

Other notifications you may see

- You may also see a notification like this when you are using other apps on your phone. If this occurs, you can select either of the first two options ("Close app" or "Wait").

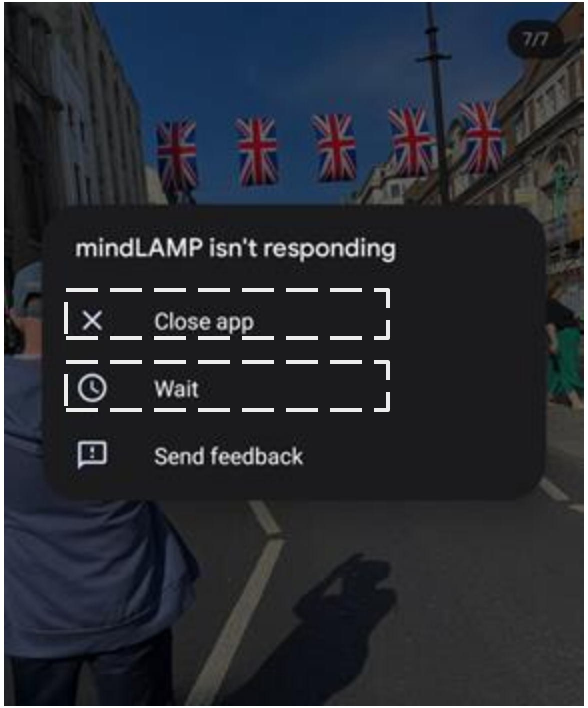

Troubleshooting Reminders

- Make sure your phone is always charged
- If your battery power is low, charge your phone

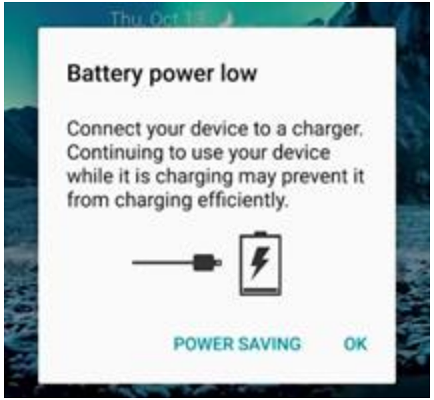

- NEVER to go into battery saver mode or airplane mode

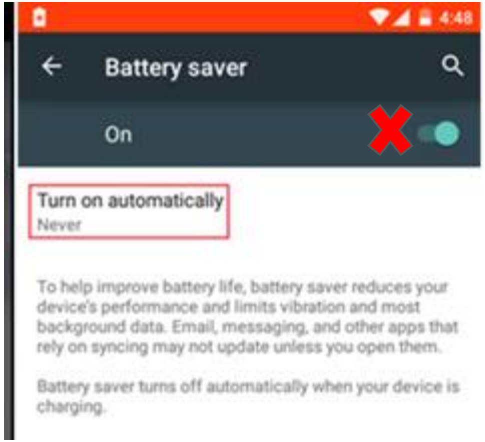

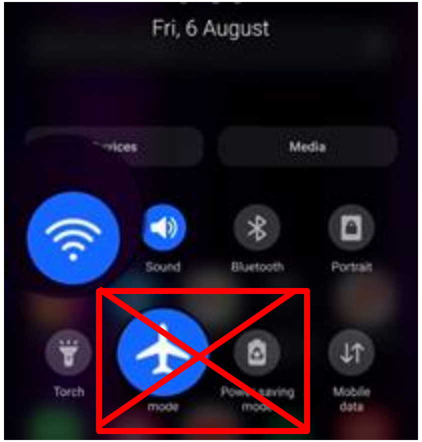

Troubleshooting Reminders

- When using the mindLAMP app, do NOT ever log out
- If you want to leave the app, simply press your home button or swipe up on your screen to exit the app

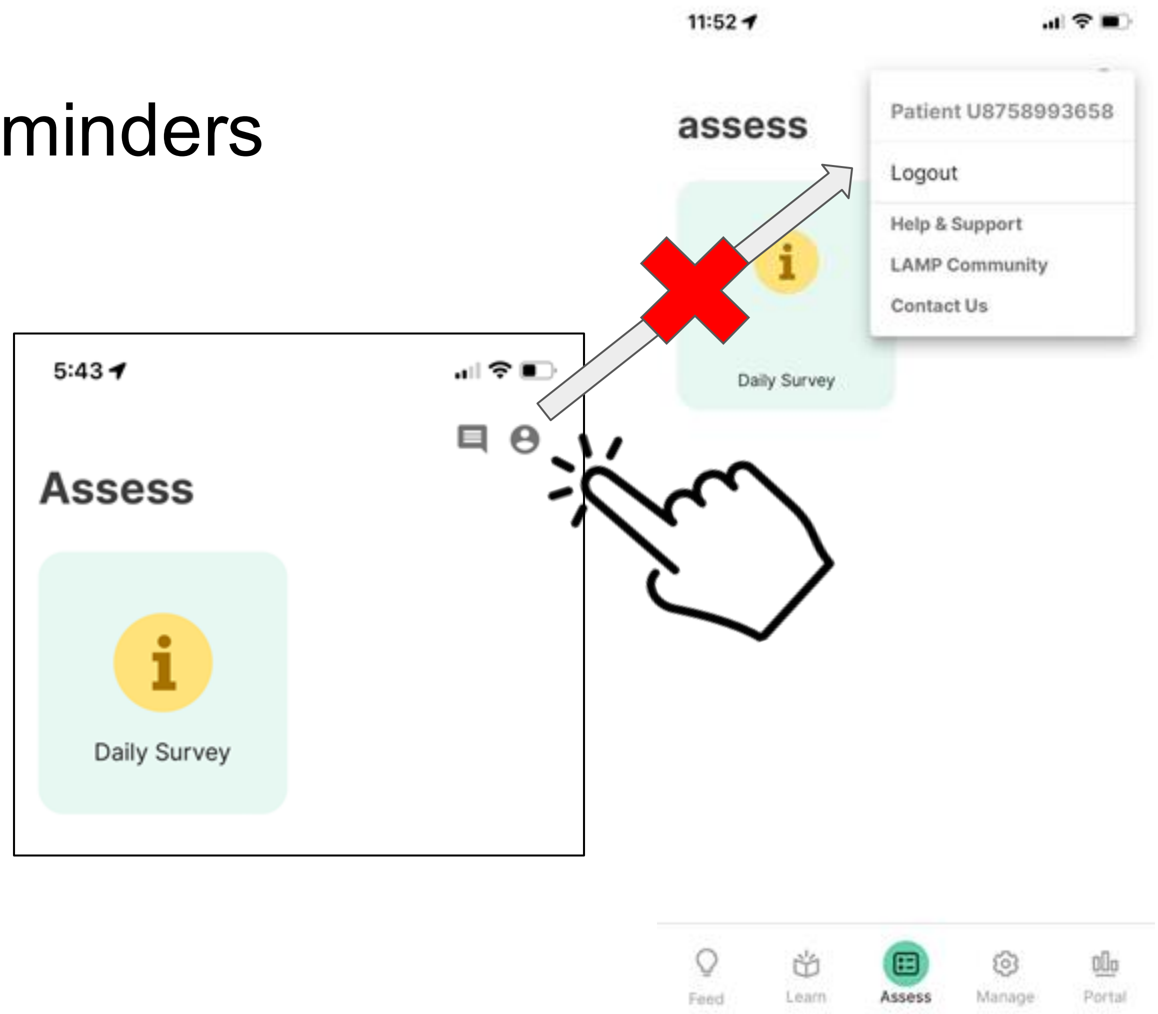

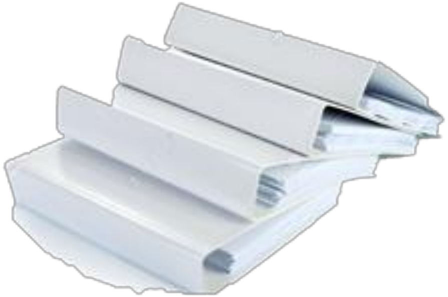

Troubleshooting Steps

- ❑ Is low power mode turned off?
- ❑ Is airplane mode turned off?
- ❑ Is the device consistently connected to WiFi?
- ❑ Is the device powered on at all times of the day?
- ❑ Are all permissions granted for mindLAMP in the Settings app?
- ❑ Are you logged in to mindLAMP?
- ❑ Have you tried closing out and relaunching the app?

Look in the study binder for directions.

Still Need Help? Contact us any time.

Email: aging@temple.edu

Phone number: **(484)-843-1321**

Examiner: record end time
(once training is completed)

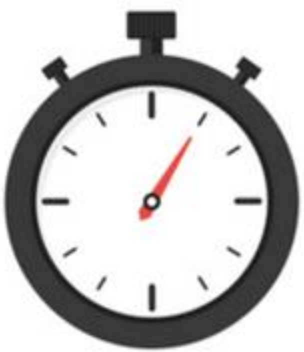

Supplement: Supplementary Material [file NIHMS2162122-supplement-Supplementary_Material.pdf]
